# Phylogeography of *Pogonomyrmex barbatus* and *P. rugosus* harvester ants with genetic and environmental caste determination

**DOI:** 10.1002/ece3.1507

**Published:** 2015-06-25

**Authors:** Brendon M Mott, Jürgen Gadau, Kirk E Anderson

**Affiliations:** 1 USDA-ARS Carl Hayden Bee Research CenterTucson, Arizona, 85719; 2 School of Life Sciences, Arizona State UniversityTempe, Arizona, 85287; 3 Center for Insect Science, University of ArizonaTucson, Arizona, 85721

**Keywords:** Mitochondrial DNA, North American deserts, phylogeography, *Pogonomyrmex*

## Abstract

We present a phylogeographic study of at least six reproductively isolated lineages of new world harvester ants within the *Pogonomyrmex barbatus* and *P. rugosus* species group. The genetic and geographic relationships within this clade are complex: Four of the identified lineages show genetic caste determination (GCD) and are divided into two pairs. Each pair has evolved under a mutualistic system that necessitates sympatry. These paired lineages are dependent upon one another because their GCD requires interlineage matings for the production of F1 hybrid workers, and intralineage matings are required to produce queens. This GCD system maintains genetic isolation among these interdependent lineages, while simultaneously requiring co-expansion and emigration as their distributions have changed over time. It has also been demonstrated that three of these four GCD lineages have undergone historical hybridization, but the narrower sampling range of previous studies has left questions on the hybrid parentage, breadth, and age of these groups. Thus, reconstructing the phylogenetic and geographic history of this group allows us to evaluate past insights and hypotheses and to plan future inquiries in a more complete historical biogeographic context. Using mitochondrial DNA sequences sampled across most of the morphospecies’ ranges in the U.S.A. and Mexico, we conducted a detailed phylogeographic study. Remarkably, our results indicate that one of the GCD lineage pairs has experienced a dramatic range expansion, despite the genetic load and fitness costs of the GCD system. Our analyses also reveal a complex pattern of vicariance and dispersal in *Pogonomyrmex* harvester ants that is largely concordant with models of late Miocene, Pliocene, and Pleistocene range shifts among various arid-adapted taxa in North America.

## Introduction

Phylogeography is an integrative field of study, drawing upon a wide array of micro- and macro-evolutionary disciplines (Avise 2000). Its aim was to elucidate the causal framework responsible for the often-observed correlation between genealogical lineages and geographic distributions. A phylogeographic approach is also appealing to biologists interested in the evolution of broadly distributed species groups because of its explicit emphasis on the two primary drivers of neutral divergence, space and time (Avise 2000).

The arid lands of the North American Southwest have long been an area of interest for traditional biogeography because of the area’s unique collection of species-rich regional deserts hemmed in by a series of largely parallel mountain ranges. The core set of regional deserts were first enumerated on the basis of plant diversity more than 60 years ago (Shreve 1942). However, the rise of modern phylogeography, facilitated by advances in sequencing technology and the discovery of rapidly evolving markers suitable for intraspecific phylogenetics, has provided new insights into the spatiotemporal patterns of divergence within the many broadly distributed species groups throughout these regions (Zink et al. 2000; Riddle and Hafner 2006a). The recent surge in these studies has also allowed for new methods of meta-analyses. Comparative phylogeography seeks to identify generalized hypotheses of endemism, dispersal, and vicariance by incorporating data from a taxonomically diverse but codistributed set of species groups (Arbogast and Kenagy 2001; Riddle and Hafner 2006b). These studies, which have primarily focused on patterns in reptiles, rodents, and birds, have not only furthered our understanding of historical vicariance events in these regions, but they have also provided keen support for the use of phylogeographic methods when investigating the evolution of any broadly distributed species group. As stated in Riddle et al. (2000a), these studies have demonstrated that “taxonomic species frequently fail to capture the inherent geographic diversity in two ways.” The first occurs because multiple divergent lineage groups are often embedded within the range of a single morphospecies, and the second failure arises because phylogenetic analyses among closely related samples often reveal that the nominal morphospecies is not monophyletic.

To date, there have been comparatively few phylogeographic studies on invertebrate taxa in this region. However, the harvester ant genus *Pogonomyrmex* contains two well-studied sister species, *P. barbatus* and *P. rugosus*, whose monophyly has been clearly challenged by recent evidence for historical hybridization and mitochondrial introgression between lineages of the two species (Helms Cahan and Keller 2003). These hybrid lineages have drawn particular interest because of their association with a unique system of genetic caste determination (GCD) found only in these two species. *P. barbatus* and *P. rugosus* are also among the most ecologically dominant and geographically widespread members of their genus, which makes them frequent study organisms in a group of seed-harvesting ants that has become famous as a model for foraging ecology (Hölldobler and Wilson 1990). Despite the intensity of study on this group, relatively little is known about the broader phylogeographic patterns of dispersal and vicariance for these or any of the other species in this genus. This makes them an ideal candidate for phylogeographic studies, as such analyses have the potential to inform both specific hypotheses on the origins and evolution of the unique GCD phenotype, as well as to provide a model for further investigations on the broader evolutionary patterns of the genus and similarly distributed invertebrate taxa throughout the arid southwest.

Taxonomists studying the morphology of *Pogonomyrmex* have long detected patterns of hybridization and noted significant intraspecific variation across the broad distributions of some species (Cole 1968). It was only within the last fifteen years, however, that researchers uncovered the molecular signals of both previous hybridization and more recent reproductive isolation among four lineages nested within the sister species complex of *P. barbatus* and *P. rugosus* (Julian et al. 2002; Volny and Gordon 2002; Helms Cahan and Keller 2003). More importantly, these newly discovered lineages were found to possess a wholly unique form of genetic caste determination (GCD), different from the common environmental caste determination (ECD) mechanisms that rely on nutritional and hormonal cues to control female (diploid) cast development in most ants (Nijhout and Wheeler 1982; Wheeler 1986; Evans and Wheeler 2001). In contrast, female brood in a GCD colony appears to have lost almost all plasticity for caste development, with a very strong correlation between genotype and caste fate (Julian et al. 2002; Volny and Gordon 2002). The workers of *P. barbatus* and *P. rugosus* are effectively sterile. Thus, the evolution of a strict genetic mechanism that always forces certain genotypes to develop into workers would seem to be unstable and presumably short-lived. The seemingly paradoxical evolution of a bias toward sterility was possible in this system because GCD lineages are always found in pairs. Queens of each paired lineage are highly polyandrous (as are the ECD lineage queens); they generate new reproductive daughters via matings with males from their own lineage, and it is only the interlineage (effectively F1 hybrid) matings that produce sterile workers (Julian et al. 2002; Volny and Gordon 2002; Helms Cahan and Keller 2003). It is their mutual dependence on an F1 interlineage workforce that necessitates sympatry for paired lineages, but because only intralineage fertilizations achieve reproductive status, each lineage within a pair remains reproductively isolated.

Two such systems of dependent lineage pairs have been described (but see Schwander et al. 2007a). Here, we refer to them as either J lineages (J1/J2) or H lineages (H1/H2) (Helms Cahan and Keller 2003). However, these lineages are morphologically cryptic: The J lineages are generally indistinguishable from the (presumed) ancestral ECD *P. barbatus*, and the H lineages are generally indistinguishable from the ancestral ECD *P. rugosus* (Anderson et al. 2006). Thus, we will adopt the nomenclature of that paper here, using the taxonomic names “*P. barbatus”* and “*P. rugosus*” to refer to nominal morphospecies in the absence of genetic data on caste determination phenotype (ECD or GCD).

Despite intensive study on the local occurrence of the J and H lineage pairs, numerous questions remain about the origins and distributions of these lineages, the latter potentially serving as an indicator of the age, success, and evolutionary stability of the system (Anderson et al. 2006). To address these questions, Anderson et al. (2006) collected allozyme data from colony samples of workers and gynes throughout the U.S. portion of the species pair’s range. Combined with a mitochondrial DNA (mtDNA) phylogeny, they were able to identify the range of the J lineages as a geographically discrete subset of the morphospecies *P. barbatus*, confined mostly to the Apache Highlands Ecoregion and with no apparent overlap between the ranges of the two groups (Fig.1 in Anderson et al. 2006). The distribution of *P. rugosus*-like H lineages showed more overlap between both ECD *P. rugosus* in the west and ECD *P. barbatus* in the east. Despite these areas of sympatry and parapatry, genetic analyses in several studies have found the J and H lineages to be reproductively isolated from ECD *P. rugosus* and *P. barbatus* (Anderson et al. 2006; Helms Cahan et al. 2006; Schwander et al. 2007a), which means they may be better described as four cryptic species.

**Figure 1:**
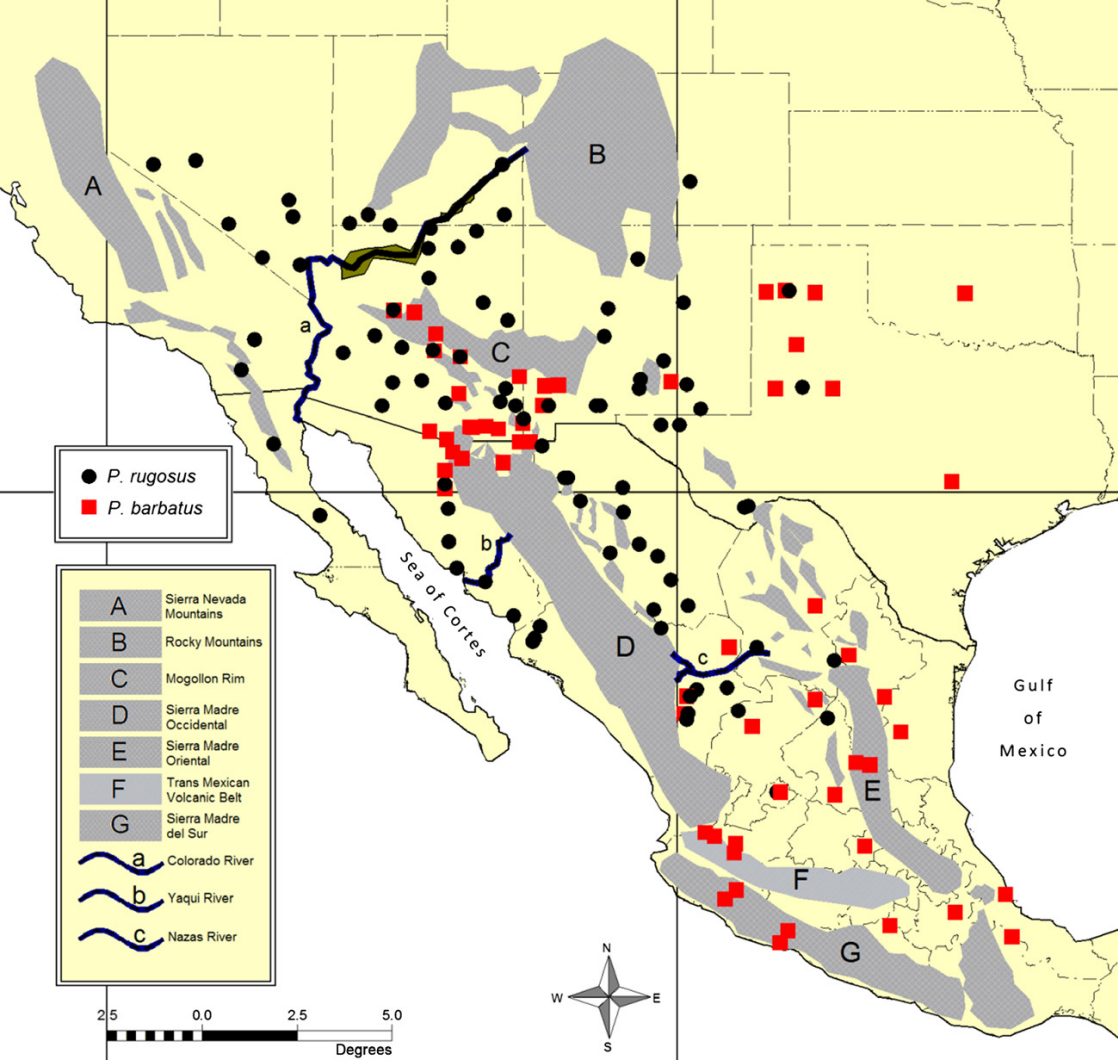
Distribution of 158 localities sampled throughout the known range of *Pogonomyrmex barbatus* and *P. rugosus* (identified by morphology only). Major mountain ranges (A–G), rivers (a–c), and bodies of water correspond to biogeographic regions and vicariance hypotheses discussed in the text.

Phylogenetic analyses in three separate studies have confirmed a pattern of historical bidirectional mitochondrial introgression among the J and H lineages, which is among the most conspicuous lines of evidence for ancestral hybridization (Helms Cahan and Keller 2003; Anderson et al. 2006; Schwander et al. 2007a). The J1 lineage samples are confined to a single monophyletic clade nested within the ECD *P. rugosus* branch of the tree, indicating that they possess an introgressed mitochondrial haplotype. Similarly, both H lineages seem to possess introgressed mitochondria, as their sequences are confined to a single monophyletic clade, rooted by the J2 lineage and nested within what has been identified as the broader ECD *P. barbatus* set of haplotypes (Fig.2 in Anderson et al. 2006).

**Figure 2:**
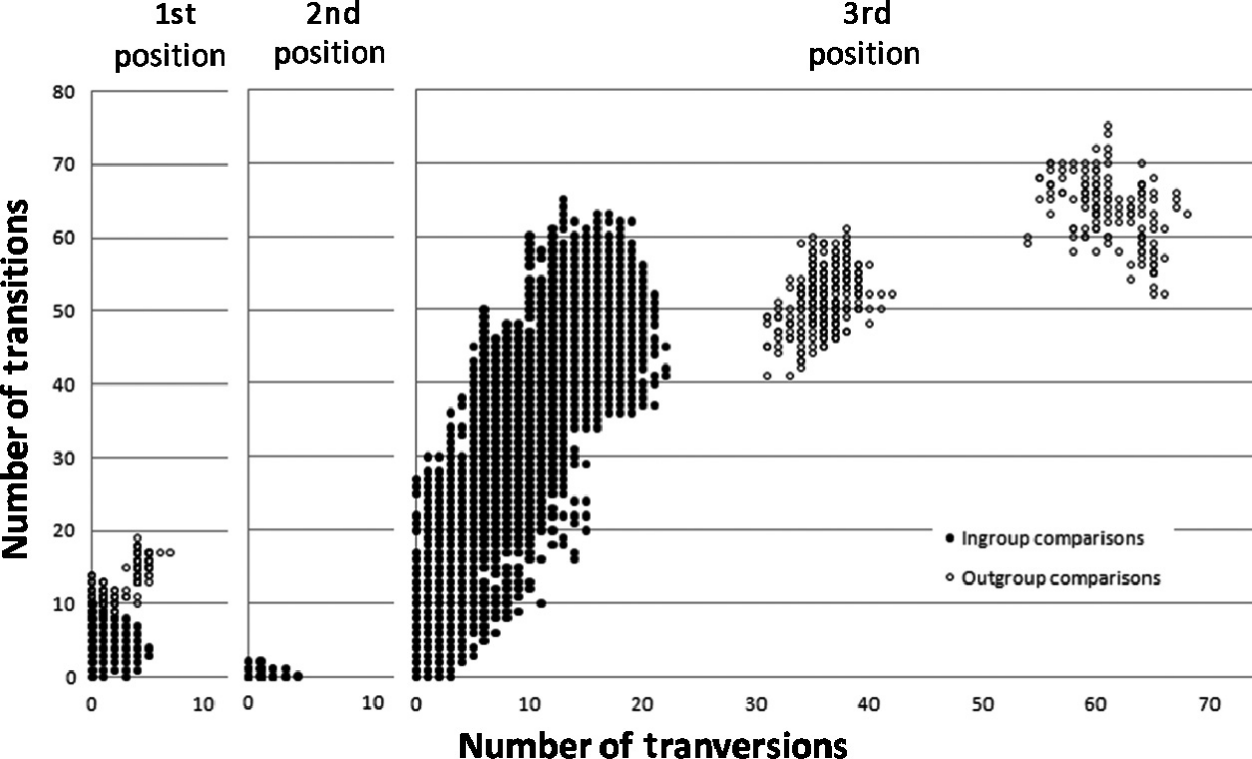
Visual depiction of evolutionary rates across the three codon positions, as shown by plotting the number of transitions versus the number of transversions for all pairwise comparisons in the 161 sequence alignment.

One hypothesis for the evolution of this unique system has focused on the evidence for historical hybridization in the J and H lineages, suggesting that the GCD system’s loss of developmental plasticity for caste development occurred as a direct result of genetic reshuffling among hybrids of the two species (Helms Cahan and Keller 2003). However, Anderson et al. (2006) disputed the characterization of the J2 lineage as a hybrid, noting that only the other three GCD lineages (J1, H1, and H2) showed a clear pattern of hybrid introgression. Additionally, Anderson et al. (2006) argued that the patterns of mtDNA divergence among the four GCD lineages were inconsistent with a hypothesis of a single hybrid origin because the J2 lineage was much more highly diverged than the others, indicating that it may have been much older. This led to the hypothesis that the unique GCD system may have evolved in the ancestors of the J2 lineage first, before the noted hybridization events occurred (Anderson et al. 2006). This hypothesis posits that the GCD phenotype may have initially spread as a self-selecting egoistic gene system, which may have secondarily introgressed into the J1 and H lineages (Anderson et al. 2006) before they reached their current (apparent) stability as reproductively isolated lineages.

One additional line of evidence comes from a key *P. barbatus* sample from southern Mexico, near the limit of the species’ southern range. The MX2 sample has an unknown caste phenotype, but it occupies a basal position within the J2 clade in the phylogeny of Anderson et al. (2006). This raised the possibility that the J2 lineage might extend through central Mexico, perhaps with some form of ancestral GCD that did not include J1, and such a finding would add support to the hypothesis that the unique caste determination system may be older than the hybrid J1 and H lineages (Anderson et al. 2006). Despite continued study in the U.S. portion of their range, this debate remains unresolved. It will likely prove intractable until the genetic architecture guiding GCD brood development is discovered. However, researchers on both sides of the debate have pointed to the need for a more thorough geographic sampling of the species complex, with an emphasis on the vastly understudied portions of its range throughout the arid lands of Mexico (Anderson et al. 2006; Schwander et al. 2007a).

Here, we present a broad phylogeographic study of the nominal morphospecies *P. barbatus* and *P. rugosus* using mitochondrial gene sequences. As far as the authors are aware, this is among the first geographically complete phylogeographic studies for any group within the genus, and perhaps the first for any native ant in North America. This means that very little is known about either the age or geographic shape of the ancestral lineage-sorting events that eventually gave rise to these two species. Fortunately, there has been a great deal of study on arid-adapted vertebrate species throughout western North America, so we are able to frame our predictions according to several established vicariance paradigms for the region. Where the current distributions of *P. barbatus* and *P. rugosus* reflect a relatively stable and ancient range from the late Miocene to Pliocene, we should expect to find broad patterns of isolation coinciding with tectonic events that formed the major mountain ranges and other geologic transformations that underlay the early formation of regional deserts (Riddle and Hafner 2006b). Specifically, intraspecific distributions may show broad patterns of east–west division along three major north–south physiographic barriers (Sea of Cortes, Sierra Madre Occidental, and Sierra Madre Oriental, Fig.1). However, the more recent climatic oscillations of the Pleistocene caused repeated episodes of desert expansion and contraction, and these processes may also have led to fragmentation as groups became isolated in separate refugia (Riddle and Hafner 2006b). These hypothetical desert refugia are less well characterized, but we might expect shallow (i.e., more recent) north–south divisions along the Baja Peninsula (Riddle et al. 2000a), the Sonoran–Sinaloan transition zone (Hafner and Riddle 2005), and along the Río Nazas on the Mexican Altiplano (Hafner et al. 2008). Similarly, we might expect east–west divisions between the Mojave and Sonoran deserts (near the course of the Colorado River) and between the Sonoran and Chihuahuan deserts, which meet at the border of Arizona, New Mexico, and Mexico (Riddle and Hafner 2006b). Finally, there is the potential for lineages to span across multiple regional deserts, which is generally interpreted as evidence for very recent expansion after the last glacial retreat ∼11,000 years ago (Riddle and Hafner 2006b).

With respect to the distribution of the J and H lineages, nothing is known about the extent to which they may inhabit as-yet unsampled portions of their morphospecies’ respective ranges. The phylogeny reported in Anderson et al. (2006) included a single *P. barbatus* sample from Mexico that was nested within the J2 clade of GCD *P. barbatus*. This suggests that the J2 clade (and presumably the J1 clade with it) may extend deep into Mexico across the Chihuahuan Desert. Likewise, nothing is known about the distribution of the H lineages in Mexico. If either the J or H lineages, or a closely related sister group, are found in central Mexico, then this could indicate that Mexican populations of *P. rugosus* and *P. barbatus* are better suited for studying their hybrid ancestry. This makes further investigation of this region critical for any inference on the origins of the GCD phenotype. It is also noteworthy that the other Mexican *P. barbatus* sample included in Anderson et al. (2006) appeared to be closely related with the ECD *P. barbatus* found far to the north in Texas and New Mexico. This may indicate a broader pattern of east–west division within the *P. barbatus* species, possibly corresponding to the Sierra Madre Oriental as outlined above.

## Materials and Methods

### Distribution mapping and sample collection

This study includes collections from a large number of focused transects, the sum of which covers the majority of each species’ known range (Cole 1968; Johnson 2000a). These transects were designed to achieve two primary goals. The first was to acquire a broad range of roughly evenly distributed samples, allowing us to produce a more complete picture of lineage differentiation across the combined range of the two species. The second goal was to refine our estimates of each species respective distributions, with an emphasis on demarcating subgroup boundaries (if present), as well as areas of transition and sympatry between species and lineages. These focal areas were predicted from the published distribution maps of the two morphospecies, and from arid habitat transition zones reported in broad biogeographic studies (e.g., Riddle and Hafner 2006b; Brown and Brennan 2007). In addition, we also collected qualitative data on the density and apparent continuity of local and regional scale distributions for each morphospecies. This process was constrained by time and limited road accessibility in various remote regions, and a thorough update to the published distributions of these species is beyond the scope of this study. However, we will refer to these observations in the Results and Discussion, including several tentative suggestions for amendments to the published distributions.

Our phylogenetic analyses include 158 single worker samples, including 111 new collections and 47 sequences from a previous study (Anderson et al. 2006) (See Table1 for detailed summary). Each worker sample represents a single population from one of 141 discrete geographic sites, covering most of the known range of the two species (Fig.1). Seventeen of the 141 sites were identified a priori as areas of sympatry between two lineages; thus, each of these sites was included twice, with one sample for each lineage. Following the descriptions for *P. barbatus* and *P. rugosus* by Cole (1968), a combination of head and thorax sculpture, as well as color, was used to assign samples from each site to either of two nominal morphospecies. Because there is often considerable variation in these traits within and among neighboring populations, multiple collections from multiple colonies were used (where available) to assign morphospecies despite there being only a single sample included for molecular analysis.

**Table 1:** Detailed summary of all sample collections and their analysis. Minor clades with highly similar taxa (≤3 bp divergence) were reduced to a single representative (bold IDs marked with a bullet •) for computationally intensive portions of the analysis.

Sequence ID	Redundant sample groups	Coordinates (degrees North, degrees West)	Morphospecies	mtDNA species	Phylogenetic clade groups		Known or inferred caste determination phenotype
					Macrogroup	Subgroup	
P. hua_AY510657	–	–	*P. huachucanus*	–	Outgroup	–	–
P. bad_AY510634	–	–	*P. badius*	–	Outgroup	–	–
P. bic_AY510644	–	–	*P. bicolor*	–	Outgroup	–	–
Pr1	–	29.3852, -114.3819	*P. rugosus*	^*^	South Prug	Baja Prug	ECD^inferred^
Pr2-G33	Grp33	31.2677, -115.5977	*P. rugosus*	^*^	North Prug	Prug 3	ECD^inferred^
Pr3-G33	Grp33	33.2152, -116.4544	*P. rugosus*	^*^	North Prug	Prug 3	ECD^inferred^
Pr4	–	34.0094, -116.0961	*P. rugosus*	^*^	North Prug	Prug 3	ECD^inferred^
**Pr5-G33•**	**Grp33•**	37.0433, -116.7700	*P. rugosus*	^*^	North Prug	Prug 3	ECD^inferred^
Pr6	–	37.6725, -115.1952	*P. rugosus*	^*^	North Prug	Prug 3	ECD^inferred^
Pr7	–	37.2294, -115.0877	*P. rugosus*	^*^	North Prug	Prug 3	ECD^inferred^
Pr8	–	37.0608, -113.5955	*P. rugosus*	^*^	North Prug	Prug 3	ECD^inferred^
**Pr9-G32•**	**Grp32•**	37.2900, -113.1186	*P. rugosus*	^*^	North Prug	Prug 3	ECD^inferred^
Pr10-G32	Grp32	37.0202, -112.5388	*P. rugosus*	^*^	North Prug	Prug 3	ECD^inferred^
Pr11	–	34.1083, -112.9402	*P. rugosus*	^*^	North Prug	Prug 3	ECD^inferred^
Pr12	–	36.9197, -111.4797	*P. rugosus*	^*^	North Prug	Prug 1	ECD^inferred^
Pr13-G26	Grp26	35.6238, -111.5169	*P. rugosus*	^*^	North Prug	Prug 1	ECD^inferred^
Pr14-G26	Grp26	36.4016, -111.5333	*P. rugosus*	^*^	North Prug	Prug 1	ECD^inferred^
**Pr15-G27†**	**Grp27•**	36.4391, -110.7516	*P. rugosus*	^*^	North Prug	Prug 1	ECD^inferred^
Pr16-G26	Grp26	36.8525, -110.2691	*P. rugosus*	^*^	North Prug	Prug 1	ECD^inferred^
**Pr17-G26•**	**Grp26•**	37.2838, -109.5327	*P. rugosus*	^*^	North Prug	Prug 1	ECD^inferred^
Pr18-G26	Grp26	38.6066, -109.5872	*P. rugosus*	^*^	North Prug	Prug 1	ECD^inferred^
Pr19-G27	Grp27	34.5150, -109.4505	*P. rugosus*	^*^	North Prug	Prug 1	ECD^inferred^
Pb20-G28	Grp28	32.7819, -108.4644	*P. barbatus*	*P. rugosus*	J	J1	GCD^inferred^
Pb21-G16^†^	Grp16	32.8119, -108.1130	*P. barbatus*	^*^	East Pbar	East Pbar 1	ECD^inferred^
**Pb22-G16•^†^**	**Grp16•**	32.8119, -108.1130	*P. barbatus*	^*^	East Pbar	East Pbar 1	ECD^inferred^
**Pr23-G2•**	**Grp2•**	32.9586, -105.9547	*P. rugosus*	*P. barbatus*	H	H	GCD^inferred^
Pr24-G27	Grp27	36.1233, -106.0255	*P. rugosus*	^*^	North Prug	Prug 1	ECD^inferred^
Pr25-G4	Grp4	38.1516, -104.6502	*P. rugosus*	*P. barbatus*	H	H	GCD^inferred^
Pr26	–	31.7702, -105.4186	*P. rugosus*	*P. barbatus*	H	H	GCD^inferred^
Pr27-G4	Grp4	33.4575, -105.3380	*P. rugosus*	*P. barbatus*	H	H	GCD^inferred^
**Pr28-G4•**	**Grp4•**	31.7613, -104.9322	*P. rugosus*	*P. barbatus*	H	H	GCD^inferred^
Pr30	–	32.1769, -104.3783	*P. rugosus*	*P. barbatus*	H	H	GCD^inferred^
Pr31	–	29.5930, -103.2263	*P. rugosus*	*P. barbatus*	H	H	GCD^inferred^
Pr32-G1	Grp1	29.6233, -103.1166	*P. rugosus*	*P. barbatus*	H	H	GCD^inferred^
**Pb35-G18•**	**Grp18•**	18.9177, -97.6894	*P. barbatus*	^*^	East Pbar	East Pbar 2	ECD^inferred^
**Pb36-G21•**	**Grp21•**	18.2852, -96.1927	*P. barbatus*	^*^	East Pbar	East Pbar 2	ECD^inferred^
Pb37	–	21.0336, -104.2558	*P. barbatus*	^*^	SWest Pbar	SWest Pbar 1	ECD^inferred^
**Pb38-G13•**	**Grp13•**	20.9233, -104.0083	*P. barbatus*	^*^	SWest Pbar	SWest Pbar 1	ECD^inferred^
Pb39-G9	Grp9	20.7350, -103.4491	*P. barbatus*	^*^	SWest Pbar	SWest Pbar 2	ECD^inferred^
Pb40	–	20.4927, -103.4902	*P. barbatus*	^*^	SWest Pbar	SWest Pbar 2	ECD^inferred^
Pb41-G13	Grp13	19.5072, -103.4427	*P. barbatus*	^*^	SWest Pbar	SWest Pbar 1	ECD^inferred^
Pb42	–	19.2650, -103.7247	*P. barbatus*	^*^	SWest Pbar	SWest Pbar 1	ECD^inferred^
Pb43-G12	Grp12	18.1188, -102.2811	*P. barbatus*	^*^	SWest Pbar	SWest Pbar 1	ECD^inferred^
**Pb44-G12•**	**Grp12•**	18.4352, -102.0875	*P. barbatus*	^*^	SWest Pbar	SWest Pbar 1	ECD^inferred^
Pr46-G3	Grp3	30.1216, -106.4188	*P. rugosus*	*P. barbatus*	H	H	GCD^inferred^
Pr47-G1	Grp1	29.4736, -106.4052	*P. rugosus*	*P. barbatus*	H	H	GCD^inferred^
Pr48	–	28.5986, -105.9911	*P. rugosus*	*P. barbatus*	H	H	GCD^inferred^
Pr49	–	28.2977, -105.5077	*P. rugosus*	*P. barbatus*	H	H	GCD^inferred^
Pr50	–	27.6591, -105.1500	*P. rugosus*	*P. barbatus*	H	H	GCD^inferred^
Pr51-G1	Grp1	26.8897, -105.6011	*P. rugosus*	*P. barbatus*	H	H	GCD^inferred^
Pr52	–	26.3994, -105.4141	*P. rugosus*	*P. barbatus*	H	H	GCD^inferred^
Pr54	–	24.7866, -104.4772	*P. rugosus*	*P. barbatus*	H	H	GCD^inferred^
**Pb55-G10•^†^**	**Grp10•**	24.6080, -104.6447	*P. barbatus*	^*^	SWest Pbar	SWest Pbar 1	ECD^inferred^
Pr56-G24^†^	Grp24	24.6080, -104.6447	*P. rugosus*	^*^	South Prug	Basal Prug	ECD^inferred^
Pb57^†^	–	24.1525, -104.7066	*P. barbatus*	^*^	SWest Pbar	SWest Pbar 1	ECD^inferred^
Pr58^†^	–	24.1525, -104.7066	*P. rugosus*	^*^	South Prug	Basal Prug	ECD^inferred^
**Pr59-G24•**	**Grp24•**	23.9941, -104.7358	*P. rugosus*	^*^	South Prug	Basal Prug	ECD^inferred^
Pr60	–	26.0572, -108.7805	*P. rugosus*	^*^	South Prug	S.Mx Prug	ECD^inferred^
Pr61	–	26.1286, -108.7388	*P. rugosus*	^*^	South Prug	S.Mx Prug	ECD^inferred^
**Pr62-G25•**	**Grp25•**	26.4430, -108.6008	*P. rugosus*	^*^	South Prug	S.Mx Prug	ECD^inferred^
Pr64-G25	Grp25	26.7266, -109.2850	*P. rugosus*	^*^	South Prug	S.Mx Prug	ECD^inferred^
Pr66-G25	Grp25	27.6055, -110.0430	*P. rugosus*	^*^	South Prug	S.Mx Prug	ECD^inferred^
Pr67	–	27.9783, -110.7788	*P. rugosus*	^*^	South Prug	S.Mx Prug	ECD^inferred^
Pr68	–	28.6683, -110.9958	*P. rugosus*	^*^	North Prug	Prug 2	ECD^inferred^
Pr69	–	29.5544, -111.0130	*P. rugosus*	^*^	North Prug	Prug 2	ECD^inferred^
Pb70	–	30.0811, -111.0908	*P. barbatus*	*P. rugosus*	J	J1	GCD^inferred^
Pr71	–	30.2025, -111.0919	*P. rugosus*	^*^	North Prug	Prug 2	ECD^inferred^
Pb72-G5	Grp5	30.5641, -111.0913	*P. barbatus*	^*^	J	J2	GCD^inferred^
Pr97-G29	Grp29	32.2685, -112.7393	*P. rugosus*	^*^	North Prug	Prug 2	ECD^inferred^
Pr99	–	32.8848, -112.4687	*P. rugosus*	^*^	North Prug	Prug 2	ECD^inferred^
**Pr100-G29•**	**Grp29•**	32.3371, -111.0826	*P. rugosus*	^*^	North Prug	Prug 2	ECD^inferred^
J2-1-G5^†^	Grp5	30.8865, -110.6374	*P. barbatus*	^*^	J	J2	GCD^1^
J2-2^†^	–	31.7347, -110.0180	*P. barbatus*	^*^	J	J2	GCD^1^
**J2-3-G6•^†^**	**Grp6•**	31.3151, -108.8500	*P. barbatus*	^*^	J	J2	GCD^1^
J2-4^†^	–	30.7830, -109.5761	*P. barbatus*	^*^	J	J2	GCD^1^
**J2-5-G5•^†^**	**Grp5•**	32.5863, -110.7250	*P. barbatus*	^*^	J	J2	GCD^1^
**J2-6-G7•^†^**	**Grp7•**	31.7044, -110.4402	*P. barbatus*	^*^	J	J2	GCD^1^
J2-7^†^	–	31.5886, -111.4961	*P. barbatus*	^*^	J	J2	GCD^1^
J2-8-G5^†^	Grp5	33.0369, -109.1311	*P. barbatus*	^*^	J	J2	GCD^1^
J1-1^†^	–	30.8865, -110.6374	*P. barbatus*	*P. rugosus*	J	J1	GCD^1^
**J1-2-G28•^†^**	**Grp28•**	31.7347, -110.0180	*P. barbatus*	*P. rugosus*	J	J1	GCD^1^
**J1-3-G30•^†^**	**Grp30•**	31.3151, -108.8500	*P. barbatus*	*P. rugosus*	J	J1	GCD^1^
J1-4-G30^†^	Grp30	30.7830, -109.5761	*P. barbatus*	*P. rugosus*	J	J1	GCD^1^
J1-5-G28^†^	Grp28	32.5863, -110.7250	*P. barbatus*	*P. rugosus*	J	J1	GCD^1^
J1-6-G28^†^	Grp28	31.7044, -110.4402	*P. barbatus*	*P. rugosus*	J	J1	GCD^1^
J1-7-G28^†^	Grp28	31.5886, -111.4961	*P. barbatus*	*P. rugosus*	J	J1	GCD^1^
J1-8-G28^†^	Grp28	33.0369, -109.1311	*P. barbatus*	*P. rugosus*	J	J1	GCD^1^
Pb401-G5	Grp5	31.0483, -110.8942	*P. barbatus*	^*^	J	J2	GCD^inferred^
Pb404-G6	Grp6	31.3151, -109.1387	*P. barbatus*	^*^	J	J2	GCD^inferred^
Pr405-G2	Grp2	31.2096, -108.5401	*P. rugosus*	*P. barbatus*	H	H	GCD^inferred^
Pr408red-G1^†^	Grp1	30.3752, -107.9603	*P. rugosus*	*P. barbatus*	H	H	GCD^inferred^
Pr408black-G3^†^	Grp3	30.3752, -107.9603	*P. rugosus*	*P. barbatus*	H	H	GCD^inferred^
**Pr410-G3•**	**Grp3•**	29.7603, -107.5325	*P. rugosus*	*P. barbatus*	H	H	GCD^inferred^
Pr414-G3	Grp3	28.3810, -106.7566	*P. rugosus*	*P. barbatus*	H	H	GCD^inferred^
**Pr418-G1•**	**Grp1•**	26.9952, -104.7029	*P. rugosus*	*P. barbatus*	H	H	GCD^inferred^
Pb419-G17	Grp17	25.8937, -103.6280	*P. barbatus*	^*^	East Pbar	East Pbar 1	ECD^inferred^
Pr424	–	24.8239, -103.6790	*P. rugosus*	^*^	South Prug	Basal Prug	ECD^inferred^
**Pr425-G17•**	**Grp17•**	24.2303, -103.3790	*P. rugosus*	*P. barbatus*	East Pbar	East Pbar 1	ECD^inferred^
Pb426-G10	Grp10	23.8157, -103.0091	*P. barbatus*	^*^	SWest Pbar	SWest Pbar 1	ECD^inferred^
Pr429^†^	–	22.0806, -102.2777	*P. rugosus*	*P. barbatus*	H	H	GCD^inferred^
**Pb429-G9•^†^**	**Grp9•**	22.0806, -102.2777	*P. barbatus*	^*^	SWest Pbar	SWest Pbar 2	ECD^inferred^
Pb433	–	22.0110, -100.8394	*P. barbatus*	^*^	SWest Pbar	SWest Pbar 2	ECD^inferred^
Pb436	–	22.8663, -100.2821	*P. barbatus*	^*^	Basal Pbar	Basal Pbar	ECD^inferred^
Pb437	–	22.8039, -99.9187	*P. barbatus*	^*^	Basal Pbar	Basal Pbar	ECD^inferred^
**Pb439-G19•**	**Grp19•**	23.6745, -99.1070	*P. barbatus*	^*^	East Pbar	East Pbar 2	ECD^inferred^
Pb441	–	24.5874, -99.5342	*P. barbatus*	^*^	SWest Pbar	SWest Pbar 2	ECD^inferred^
Pb444-G19	Grp19	25.6856, -100.4789	*P. barbatus*	^*^	East Pbar	East Pbar 2	ECD^inferred^
**Pr445-G15•**	**Grp15•**	25.5414, -100.8657	*P. rugosus*	*P. barbatus*	Basal Pbar	Basal Pbar	ECD^inferred^
Pr451-G15	Grp15	24.0266, -101.0435	*P. rugosus*	*P. barbatus*	Basal Pbar	Basal Pbar	ECD^inferred^
Pb453	–	24.5245, -101.3684	*P. barbatus*	^*^	SWest Pbar	SWest Pbar 2	ECD^inferred^
Pb457	–	26.9911, -101.3665	*P. barbatus*	^*^	East Pbar	East Pbar 1	ECD^inferred^
Pr462	–	25.8863, -102.9027	*P. rugosus*	*P. barbatus*	H	H	GCD^inferred^
**PbQ1-G11•^†^**	**Grp11•**	20.6663, -100.0706	*P. barbatus*	^*^	SWest Pbar	SWest Pbar 1	ECD^2^
PbQ2-G11^†^	Grp11	20.6663, -100.0707	*P. barbatus*	^*^	SWest Pbar	SWest Pbar 1	ECD^2^
MX1-G21	Grp21	19.3946, -96.3617	*P. barbatus*	^*^	East Pbar	East Pbar 2	ECD^inferred^
MX2	–	18.5697, -99.4016	*P. barbatus*	^*^	MX2	MX2	ECD^inferred^
1BAR	–	34.7666, -112.4333	*P. barbatus*	*P. rugosus*	J	J1	GCD^3^
2BAR	–	34.7166, -111.9000	*P. barbatus*	^*^	J	J2	GCD^3^
3BAR	–	34.1666, -111.3333	*P. barbatus*	*P. rugosus*	J	J1	GCD^3^
4BAR-G28	Grp28	33.7166, -111.3666	*P. barbatus*	*P. rugosus*	J	J1	GCD^3^
5BAR	–	33.5500, -110.7000	*P. barbatus*	^*^	J	J2	GCD^3^
6BAR-G8	Grp8	31.3833, -111.0500	*P. barbatus*	^*^	J	J2	GCD^3^
**7BAR-G8•**	**Grp8•**	31.7000, -110.3666	*P. barbatus*	^*^	J	J2	GCD^3^
8BAR-G7	Grp7	31.6666, -109.6833	*P. barbatus*	^*^	J	J2	GCD^3^
9BAR-G28	Grp28	31.8000, -109.0500	*P. barbatus*	*P. rugosus*	J	J1	GCD^1^
10BAR	–	32.2666, -108.5333	*P. barbatus*	^*^	J	J2	GCD^3^
11BAR^†^	–	32.2666, -108.3666	*P. barbatus*	*P. rugosus*	J	J1	GCD^3^
12BAR	–	32.9000, -105.1500	*P. barbatus*	^*^	East Pbar	East Pbar 1	ECD^3^
13BAR	–	35.2500, -102.6500	*P. barbatus*	^*^	East Pbar	East Pbar 1	ECD^3^
14BAR^†^	–	35.2833, -102.0500	*P. barbatus*	^*^	East Pbar	East Pbar 1	ECD^3^
15BAR	–	35.2333, -101.3666	*P. barbatus*	^*^	East Pbar	East Pbar 1	ECD^3^
**16BAR-G20•**	**Grp20•**	35.2166, -97.4166	*P. barbatus*	^*^	East Pbar	East Pbar 2	ECD^3^
17BAR-G20	Grp20	33.8666, -101.8500	*P. barbatus*	^*^	East Pbar	East Pbar 2	ECD^3^
18BAR-G20	Grp20	32.7166, -102.4166	*P. barbatus*	^*^	East Pbar	East Pbar 2	ECD^3^
19BAR-G18	Grp18	32.7166, -100.9000	*P. barbatus*	^*^	East Pbar	East Pbar 2	ECD^3^
20BAR	–	30.2666, -97.7666	*P. barbatus*	^*^	East Pbar	East Pbar 2	ECD^3^
**21RUG-G34•**	**Grp34•**	38.6166, -118.7666	*P. rugosus*	^*^	North Prug	Prug 3	ECD^3^
22RUG-G34	Grp34	38.7166, -117.6500	*P. rugosus*	^*^	North Prug	Prug 3	ECD^3^
23RUG	–	36.1666, -115.8833	*P. rugosus*	^*^	North Prug	Prug 3	ECD^3^
**24RUG-G31•**	**Grp31•**	35.9666, -114.9000	*P. rugosus*	^*^	North Prug	Prug 3	ECD^3^
25RUG	-	33.6666, -113.7666	*P. rugosus*	^*^	North Prug	Prug 3	ECD^3^
26RUG-G31	Grp31	34.7833, -112.4500	*P. rugosus*	^*^	North Prug	Prug 3	ECD^3^
27RUG	–	33.8000, -112.2333	*P. rugosus*	^*^	North Prug	Prug 3	ECD^3^
28RUG	–	32.9333, -111.7000	*P. rugosus*	^*^	North Prug	Prug 2	ECD^3^
29RUG	–	33.7333, -111.4166	*P. rugosus*	^*^	North Prug	Prug 3	ECD^3^
**30RUG-G23•**	**Grp23•**	33.5500, -110.7000	*P. rugosus*	^*^	North Prug	Prug 1	ECD^3^
31RUG-G27	Grp27	34.9833, -110.0833	*P. rugosus*	^*^	North Prug	Prug 1	ECD^3^
32RUG-G23	Grp23	32.7166, -109.5000	*P. rugosus*	^*^	North Prug	Prug 1	ECD^3^
33RUG	–	32.3666, -109.6333	*P. rugosus*	^*^	North Prug	Prug 2	ECD^3^
34RUG-G2	Grp2	32.2666, -109.2333	*P. rugosus*	*P. barbatus*	H	H	GCD^3^
43RUG1-G2^†^	Grp2	32.2666, -107.0166	*P. rugosus*	*P. barbatus*	H	H	GCD^3^
36RUG-G3	Grp3	31.9166, -109.0333	*P. rugosus*	*P. barbatus*	H	H	GCD^3^
37RUG^†^	–	32.2666, -108.3666	*P. rugosus*	*P. barbatus*	H	H	GCD^3^
38RUG	–	34.1000, -106.9166	*P. rugosus*	^*^	North Prug	Prug 1	ECD^3^
**39RUG-G22•**	**Grp22•**	34.8166, -106.8000	*P. rugosus*	^*^	North Prug	Prug 1	ECD^3^
41RUG	–	34.9833, -104.8166	*P. rugosus*	*P. barbatus*	H	H	GCD^3^
42RUG^†^	–	35.2833, -102.0500	*P. rugosus*	*P. barbatus*	H	H	GCD^3^
43RUG2-G22^†^	Grp22	32.2666, -107.0166	*P. rugosus*	^*^	North Prug	Prug 1	ECD^3^
44RUG	–	32.8166, -104.7333	*P. rugosus*	*P. barbatus*	H	H	GCD^3^
45RUG-G3	Grp3	32.7166, -105.9833	*P. rugosus*	*P. barbatus*	H	H	GCD^1^
46RUG-G1	Grp1	32.7500, -101.7000	*P. rugosus*	*P. barbatus*	H	H	GCD^3^

**Figure 3:**
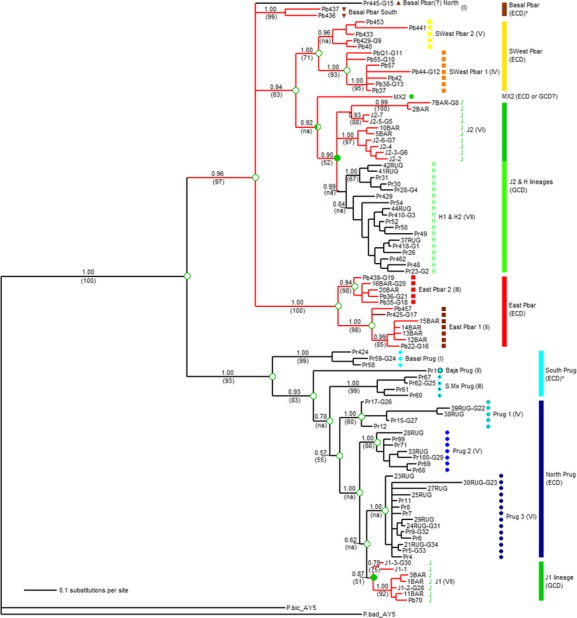
Bayesian consensus phylogram for the reduced dataset, showing inferred phylogenetic relationships among 97 samples of *Pogonomyrmex barbatus* and *P. rugosus*. Support for major branches is indicated with Bayesian posterior probabilities (above) and parsimony bootstrap values (below in parentheses). Several major clades were not recovered in the parsimony bootstrap consensus tree and are marked with (na) (see Results). Terminal sample IDs include morphospecies (or previously identified J lineage). Thirty-three sample IDs are followed by a group ID (e.g., Pr445-G15), indicating that these terminals represent two or more populations with highly similar or identical haplotypes (see Table1 and Methods). Colored bars (far right) indicate *macro*groups corresponding to geographical distributions (Fig.4). Caste determination phenotype was inferred for ancestral nodes via parsimony (indicated for major nodes with open (ECD), filled (GCD), or half-filled (undetermined) green circles) and is also listed as known or inferred* for macrogroups. Symbol columns adjacent to sample IDs refer to geographically distinct subgroups (Fig.5), and Roman numerals in parentheses refer to simplified distribution maps for each subgroup (Figs.6, 7).

**Figure 4:**
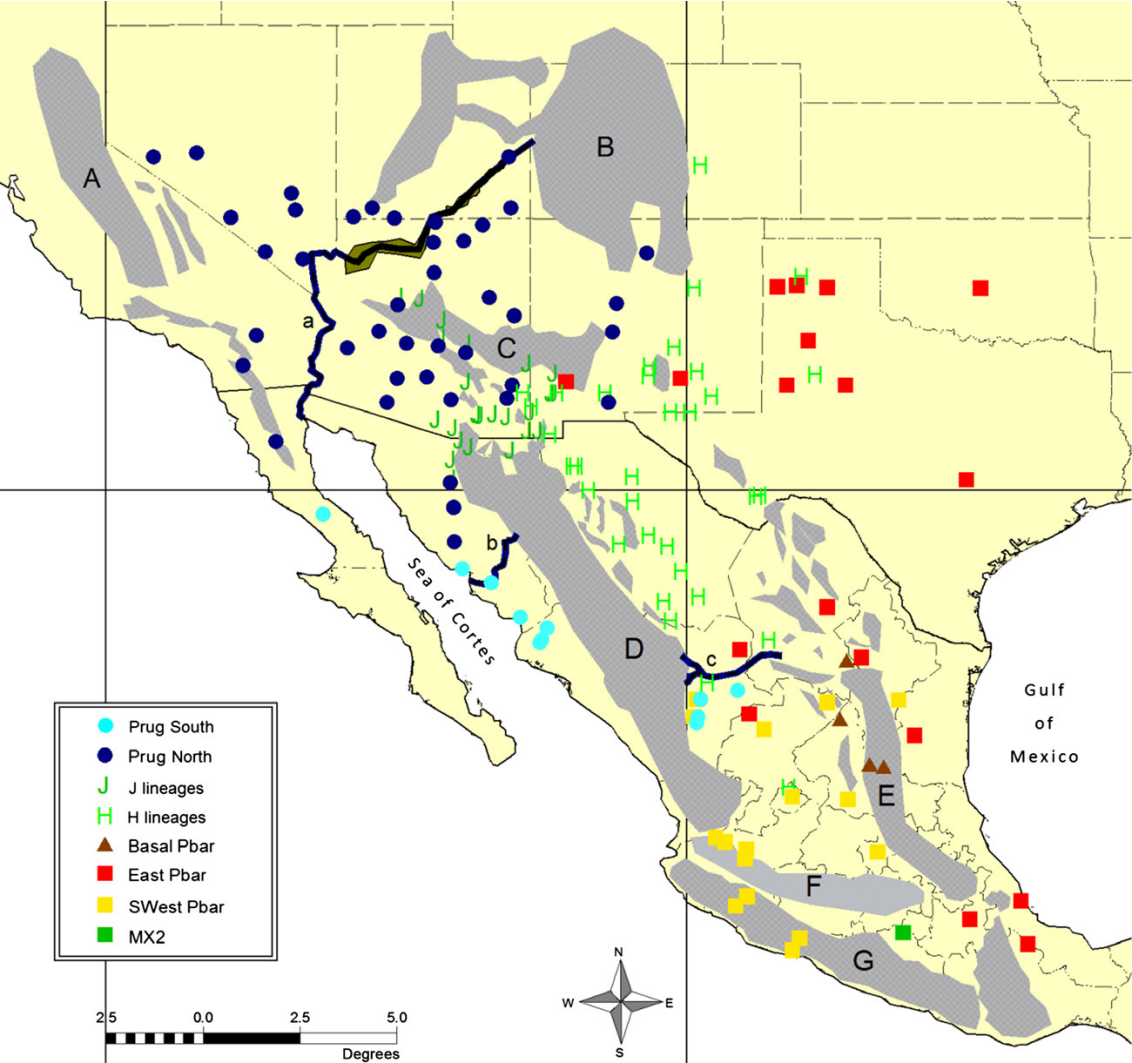
Distribution of all major macrogroup clades as identified in the phylogeny (Fig.3). Major mountain ranges (A–G) and rivers (a–c) are discussed in the text and listed in the legend for Figure1.

**Figure 5:**
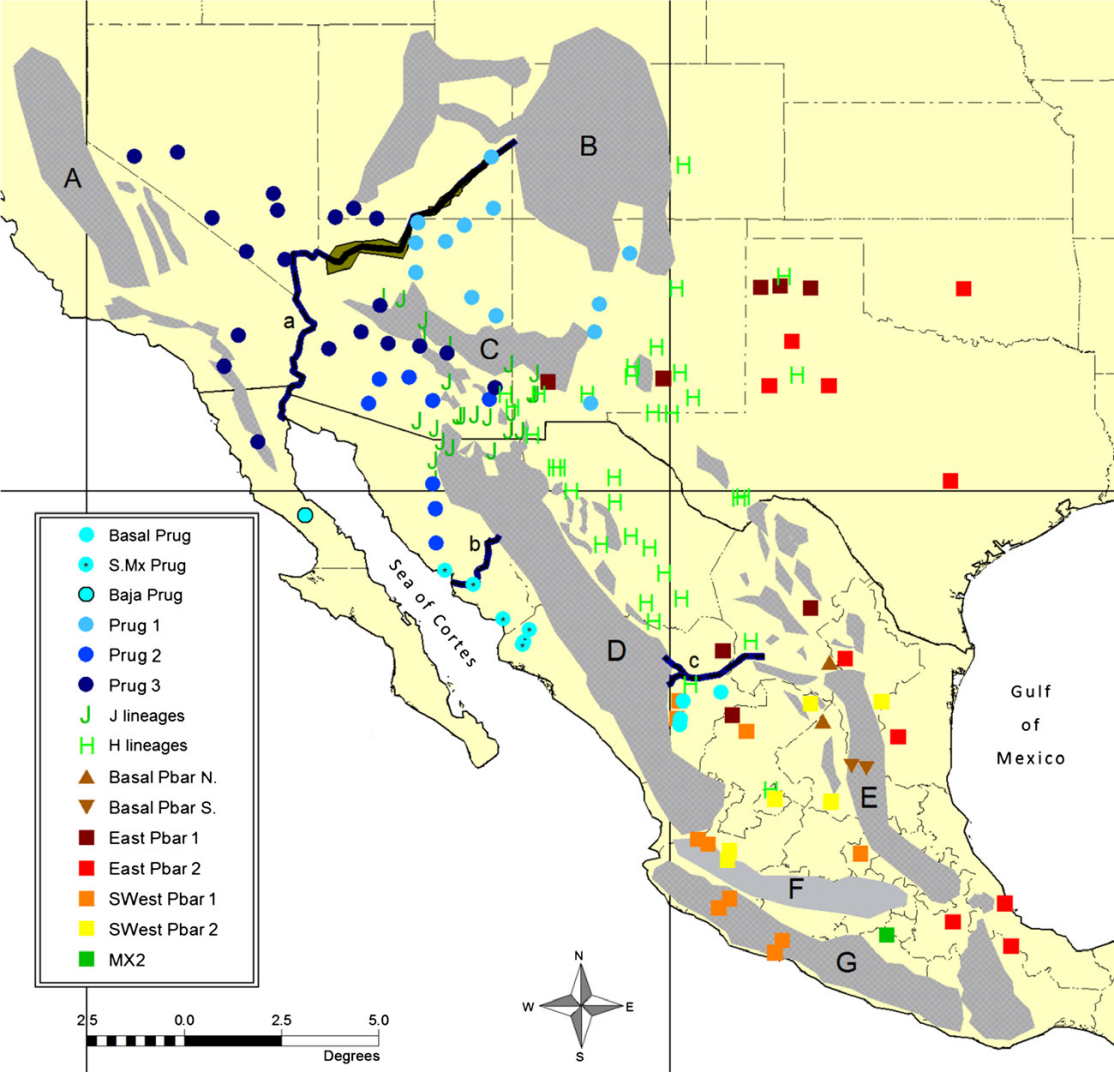
Distribution of all major subgroup clades as identified in the phylogeny (Fig.3). Major mountain ranges (A–G) and rivers (a–c) are discussed in the text and listed in the legend for Figure1.

Daggers (†) mark the 34 samples drawn from 17 sympatric sites. Morphospecies is according to Cole (1968). Ingroup samples were assigned to either of two mtDNA species categories according to the presumed species bifurcation in the phylogeny (Fig.3). Asterisks (^*^) indicate concordance between morphology and mitochondrial lineage; all other entries indicate incongruence as a result of hybrid introgression or ancestral variation. Phylogenetic clade group assignment is depicted in the phylogeny (Fig.3) and in the distribution maps (Fig.4, Fig.5). Caste determination phenotype was inferred via parsimony for new samples based on their position in the phylogeny relative to samples with known phenotypes (^1^Anderson et al. 2011, ^2^this paper; see Methods; ^3^Anderson et al. 2006).

### Molecular methods

To examine the evolutionary relationships among (mitochondrial) lineages using phylogenetic analysis, we sequenced a portion of the mitochondrial gene cytochrome *c* oxidase subunit I (*cox1*) from 111 single worker samples drawn from 97 sites. Total genomic DNA was extracted from individual workers, which had been preserved in either 95% ethanol or kept alive until transfer to a −80°C freezer, using a standard Chelex solution extraction modified from Volny and Gordon (2002). Briefly, each ant’s head and thorax were crushed with a pestle in a 1.5-mL microcentrifuge tube containing a solution of 150 *μ*L of 20% Chelex and 2 *μ*L of proteinase K (20 mg/mL). These solutions were incubated for 6–12 h at ∼57°C and then rapidly heated to ∼95°C for 5–10 min to denature the proteinase K. Finally, samples were centrifuged at high speed for 15 min, and the DNA-containing supernatant was removed. Two primer pairs were used to amplify partially overlapping regions of mitochondrial DNA (mtDNA) according to the polymerase chain reaction (PCR) methods described in Anderson et al. (2006). PCR products were purified with ExoSAP-IT according to the manufacturer’s suggested protocol (USB) and then run on an ABI 377 automated sequencer. The first primer pair, “Ben3R” (Brady et al. 2000) and “Jerry” (Simon et al. 1994), yielded an approximately 450-bp fragment after sequencing from both directions and aligning reverse complements. The second primer pair, “LCO” and “HCO” (Folmer et al. 1994), yielded more than 650 bp of sequence with both primers included. After removing redundant sites in the overlapping region and aligning our sequences to other *cox1* sequences published in GenBank, the combined fragments had a final length of 1054 bp.

### Sequence alignment and dataset assembly

Using the program Bioedit version 7.09 (Hall 1999), the 111 new sequences were manually aligned against 47 *P. barbatus* and *P. rugosus* sequences used in another study (Anderson et al. 2006). In addition to sequence length, the related issue of sequence quality can also become important when there are large amounts of missing data that may mask informative variation among samples. The 111 new sequences were of mostly high quality, with 93% of the sequences containing less than 1% missing data. However, the sequences from Anderson et al. (2006) possessed notably reduced coverage in the adjoining region of the two primer pairs, resulting in 85% of the 47 sequences containing between 3 and 5% missing data after alignment with the new data. To test for any potential confounding effects from the inclusion of these shorter sequence reads, we performed separate analyses that excluded the missing data and compared the resulting topologies (see *Distance-based analyses* below). All missing data were believed to result from sequencing limitations, so no gaps were inferred for the alignment.

Outgroup selection was potentially problematic due to several levels of taxonomic ambiguity within the genus *Pogonomyrmex*. In addition to the previously mentioned evidence for hybridization and horizontal gene transfer within our focal species pair, the broader phylogenetic relationships of the genus have also been subject to considerable debate, and recent evidence has suggested that several other species within the *P. barbatus* complex may be paraphyletic (Parker and Rissing 2002). To avoid these ambiguities, we included three progressively distal outgroup species, *P. bicolor*, *P. badius*, and *P. huachucanus* (Table1). All three of these species were identified as sister to the *P. barbatus* complex in Parker and Rissing’s study (2002).

The resulting 161-sequence alignment represented our full dataset, which was used to estimate substitution rate patterns and pairwise sequence divergence. It was also used for our preliminary phylogenetic analyses with two distance-based methods, neighbor joining (NJ) and minimum evolution (ME). The results of our initial tree searches and pairwise distance calculations revealed a large number of highly similar or identical samples that were minimally informative. These redundant samples were removed to create a reduced alignment of 99 sequences, which was employed for our primary phylogenetic analyses with the more computationally demanding character-based methods of maximum parsimony (MP) and maximum likelihood (inferred through Bayesian analysis).

Monophyletic clades (as identified by NJ and ME criterion) that contained redundant samples (≤3-bp divergence) were grouped, and a single representative for each group was randomly chosen after eliminating group members with inferior sequence quality. A total of 33 redundant sample groups were identified (Table1), and 61 samples were pruned from the full dataset. In addition, the furthest removed outgroup sample, *P. huachucanus*, was also removed, leaving the reduced alignment at 99 sequences. This condensed dataset allowed us to focus our primary analyses on the deeper clade relationships that were of interest for this study, rather than diverting computational effort toward the shallow nodes, which are in any case better addressed with other methods (Posada and Crandall 2001). Furthermore, a case study by Milinkovitch et al. (1996) emphasized that the inclusion of large numbers of “redundant” taxa can be ineffective, or even deleterious, when conducting tree searches under character based methods such as maximum parsimony and maximum likelihood.

### Preliminary (distance-based) phylogenetic analyses and model selection

Initial analyses on the full (161-sequence) dataset were employed to achieve three preliminary goals, and their results provided insight that informed the design of our primary phylogenetic analyses. First, the program MEGA 4.04 (Tamura et al. 2007) was used to estimate patterns of nucleotide substitution and potential site saturation by calculating transition/transversion ratios across all pairwise comparisons. The data’s substitution patterns were further analyzed with Modeltest 3.07 (Posada and Crandall 1998), which used a hierarchical series of NJ trees estimated in PAUP* 4.0b10 (Swofford 2002) to select a best-fit model of evolution for the full (and later the condensed) dataset(s). In both analyses, all three criteria employed by Modeltest suggested the most complex model of sequence evolution available (general time reversible with gamma-distributed among-site rate variation and a proportion of invariant sites; GTR+I+G).

The second step in our preliminary analyses involved the use of rapid phylogenetic tree searches using both the neighbor-joining (NJ) and minimum evolution (ME) criterion in MEGA. The GTR+I+G model is not implemented in MEGA 4.04, but we selected the Maximum Composite Likelihood option as the closest approximation, with a gamma shape parameter of 0.6757 as estimated by the Akaike information criterion (AIC) in Modeltest. The results from these searches were summarized with 50% majority-rule consensus trees (generated from 1000–2000 pseudo-replicate bootstraps), which were used to compare estimates of support for major branches in the recovered topologies. These trees led to the creation of the reduced (99-sequence) dataset as described above.

The last of these preliminary tests was a sensitivity analysis, designed to evaluate topological stability under various parameter options. To test for possible effects from the sequences with missing data, each NJ and ME run was repeated with both pairwise and complete deletion options for missing sites, the latter of which reduced the dataset to a 907-bp alignment with zero missing sites for all sequences. To test for any bias introduced by condensing our dataset, all of the aforementioned analyses were repeated on both the full (161-sequence) and the reduced (99-sequence) dataset.

### Primary phylogenetic analyses

The reduced (99-sequence) dataset was examined under the maximum-parsimony criterion as implemented in PAUP* 4.0b10 (Swofford 2002), with all positions and nucleotide substitutions weighted equally. We used a heuristic tree search using 100 replicate random stepwise additions with a maximum search length of 1000 sec per replicate, and the tree bisection and reconnection branch swapping algorithm, to construct our initial set of most parsimonious trees. Branch support was estimated with a similar tree search for each of 1000 bootstrap pseudo-replicates, except that only 10 stepwise addition replicates were used per bootstrap, and the search length for each of those replicates was reduced to 100 sec. These results were then used to construct a 50% majority-rule consensus tree. A second parsimony analysis on the full (161 sequence) alignment was used to test for bias introduced by the construction of our reduced dataset. For efficiency, this analysis used very restrictive search limits (10 sec per replicate, 100 replicates), and it did not include bootstrapping to measure support.

The reduced dataset was also analyzed with the maximum-likelihood (ML) model suggested by Modeltest (GTR+I+G) as implemented in the program MrBayes 3.1.2 (Huelsenbeck and Ronquist 2001). MrBayes uses a Metropolis-coupled MCMC (Markov chain Monte Carlo) approach to estimate both the tree topology and the parameters which best fit the data. The process samples a large number of similar topologies with roughly equal probabilities, and the frequency of a node among all such trees provides an approximation of its posterior probability, which is a measure of its statistical support (Holder and Lewis 2003). We used the default implementation of two parallel runs, each consisting of one “cold” and three “heated” chains, and the default flat priors for each parameter. Ideally, independent parallel runs converge in a region of stationarity, after which they should continue to sample the same range of equally likely topologies indefinitely. However, Bayesian analysis is a stochastic process that can become trapped at local optima, and it can be difficult to correctly identify when the chains have reached stationarity (Holder and Lewis 2003). To further ensure that individual analyses did not become fixed on local optima, we compared the results from six separate analyses, each with two parallel runs as described above. Analyses were run for 14–30 million generations with sampling every 1000th generation. In addition, three heating schemes were employed in an attempt to increase the efficiency of the Metropolis-coupled MCMC, with two runs each at temperatures of 0.15, 0.2 (the default), and 0.25. Convergence and stationarity were assessed using the standard deviation of split frequencies (SDSF), as well as the potential scale reduction factors for each parameter and plots of log likelihood versus generation, as generated within MrBayes. In addition, the program Tracer 1.4.1 (Rambaut and Drummond 2007) was used to visually inspect plots of all parameters versus generation for evidence of nonstationarity. All runs appeared to reach stationarity within the first 1–3 million generations, and the results prior to this point were discarded as “burn-in” before constructing phylograms from the remaining posterior distribution of trees. The phylograms from each independent run were then compared to assess convergence. It should be noted that a closer inspection of the log-likelihood plots and the SDSF for the initial runs, which used the default 0.2 temp parameter, revealed significant fluctuations after reaching apparent stationarity. This led us to reevaluate each run with multiple putative burn-in fractions, ranging from 10 to 75% of the total sample. This instability was less evident in other runs, but it was noted even in the latter half of the longest run (30 million generations) with the default temperature. In contrast, the runs with an increased temperature of 0.25 achieved apparent stationarity much earlier, and their SDSF values steadily decreased over time.

### Identifying geographically discrete Macro- and Subgroups for downstream analysis

To examine broad patterns of intraspecific fragmentation, the two mtDNA species clades (Fig.3) were divided into a series of seven geographically discrete macrogroups (Fig.4). Six of the macrogroups contained reciprocally monophyletic subclades that corresponded to discrete geographic distributions, so they were further divided into 16 total subgroups (Fig.5). Note that geographic discreteness was assessed among effective species (i.e., potential reproductive partners), so we ignored areas of overlap among *P. barbatus* and *P. rugosus* and the J1/J2 and H lineages when creating the macro- and subgroups as described above. Also note that some macrogroups are not monophyletic, but were lumped together as necessary outgroup assemblages.

To test for population isolation between subgroups, and regional isolation and structure between and within macrogroups, we used hierarchical AMOVAs and pairwise F_ST_s as implemented in Arlequin vers. 3.5 (Excoffier et al. 1992, Excoffier et al. 2005). All 158 ingroup samples were included in the initial analyses, but they were run separately for each mtDNA species tree to avoid inflating estimates of population subdivision with interspecific comparisons. Arlequin calculated a new distance matrix based on uncorrected p-distances and estimated significance for both types of analysis with 2024 random permutations of the data. The AMOVAs were structured with two levels as depicted in the phylogeny (Fig.3) and described above, with samples assigned to subgroups, and subgroups assigned to macrogroups. The pairwise F_ST_s were calculated only for subgroups. Due to our low sample size in certain groups (Baja Prug, MX2, Basal Pbar North, and Basal Pbar South), the AMOVAs and pairwise F_ST_s were repeated with these groups either excluded (in the case of Baja Prug and MX2) or merged (in the case of the two Basal Pbar subgroups). Because the *P. rugosus* clade is paraphyletic without J1 (as is the *P. barbatus* clade without J2 and H), we also reran the *P. rugosus* AMOVA with J1 merged into Prug North.

### Estimates of genetic diversity, divergence, and demographic history

After identifying statistically supported macrogroups and subgroups as described above (and see Results), MEGA was used to calculate uncorrected pairwise sequence divergence (p-distance) among and within all nominal macrogroups and subgroups. Although we lack key information necessary for molecular clock estimates, we used this data on average intergroup p-distances to estimate time since divergence with a calibration proposed in Quek et al. (2004, see Results and Discussion).

To test for possible changes in historical population size or selective sweeps, we calculated Tajima’s *D* (Tajima 1989, 1996), Fu’s *Fs* (Fu 1997), and mismatch distribution analyses (Rogers and Harpending 1992) for subgroups in Arlequin. Tajima’s *D* is widely used to test for deviations from neutrality: Positive values are consistent with a historical population contraction (i.e., bottleneck), and negative values are consistent with expansion (Tajima 1996). Fu’s *F*_*S*_ is also a test for deviations from neutrality, but it is considered more powerful for detecting the excess of rare haplotypes associated with recent population growth (Fu 1997; Ramos-Onsins and Rozas 2002). Statistical significance for both Tajima’s *D* and Fu’s *Fs* were estimated for each subgroup with ≥4 samples using 3,000 simulated samples as the null distribution.

Mismatch distribution analyses rely on the expectation that populations having undergone recent expansion exhibit a roughly unimodal and smooth distribution of the frequency of pairwise differences among all sequences, which contrasts with the more bimodal and ragged distributions expected for stable populations (Rogers and Harpending 1992). Arlequin 3.5 implements this test by comparing the shape of each sample’s empirical distribution against a large pool of simulated distributions to calculate the sum of squared deviations (*SSD*), where the simulated data are based on parameters estimated from that sample under a null model of demographic expansion (Schneider and Excoffier 1999; Excoffier et al. 2005). A second test of the mismatch distribution, Harpending’s Raggedness Index (*RI*), is expected to be significant if the mismatch distribution is rougher than expected for populations having undergone a sudden demographic expansion (Harpending 1994). Thus, nonsignificant *SSD* and *RI* statistics calculated in Arlequin indicate support for a model of sudden demographic expansion. Statistical significance for *SSD* and *RI* was estimated for each subgroup with ≥4 samples using 2,000 simulated samples.

Additionally, the program DnaSP 5.10.01 (Librado and Rozas 2009) was used to calculate Ramos-Onsins and Rozas *R*^2^ statistic, which has been shown to be similar to Fu’s *Fs* in its sensitivity to the genetic signal of a recent population expansion (Ramos-Onsins and Rozas 2002). Statistical significance for *R*^2^ was estimated for each subgroup with ≥4 samples using 3,000 replicate coalescent simulations in DnaSP.

### Inferring caste determination phenotype in new populations

Previous studies on the distribution of genetic caste determination (GCD) lineages in *P. barbatus* and *P. rugosus* (Anderson et al. 2006; Schwander et al. 2007a) have relied on genotypic assays of both workers and reproductive female gynes to detect the discrete pools of genetic diversity for each caste that are characteristic of the GCD system. However, the reproductive caste is only present in harvester ant colonies for a few months prior to the summer rains that initiate their mating flights, and the winged reproductives are rarely found outside the nest before the day when they first take flight. These factors, combined with the breadth of our sampling, made colony level assays for GCD unfeasible for this study. Fortunately, three previous studies have confirmed that the GCD phenotype maps onto just two discrete and apparently monophyletic clades in mtDNA phylogenies constructed from *cox1* sequences (Helms Cahan and Keller 2003; Anderson et al. 2006; Schwander et al. 2007a). Moreover, these and other studies have repeatedly found that J and H lineages are reproductively isolated from each other and from ECD *P. barbatus* and *P. rugosus* (Helms Cahan and Keller 2003; Anderson et al. 2006; Helms Cahan et al. 2006; Schwander et al. 2007a, 2007b; Schwander et al. 2008; Curry et al. 2010; Sirviö et al. 2010). To the extent that this pattern is maintained across a broader geographic range, it is possible to define samples as derived from either a GCD or ECD clade based on phylogenetic analyses. Thus, we can indirectly infer their caste determination phenotype.

The present study includes a large number of samples with a previously identified caste determination phenotype, including 45 samples characterized in Anderson et al. 2006 and another 16 samples drawn from colonies characterized in Anderson et al. 2011 (Table1). Two additional populations included in this study (PbQ1 and PbQ2) were identified as ECD through the use of microsatellite markers in workers and gynes from 10 colonies (data not shown). Using the program Mesquite 3.02 (Maddison and Maddison, 2015), we mapped these known states onto the 99 sequence phylogeny and then used the ancestral state reconstruction system to infer the status of ancestral nodes via simple parsimony (i.e., equal cost for all state changes). Note that we assume ECD is the ancestral state for the genus and both morphospecies studied here, but we did not input this assumption into the reconstruction. Thus, the reconstructed states are based solely on the phylogeny topology and the 43 known-state haplotypes included in our 99-sequence dataset (representing the 63 known-state populations described above).

## Results

### Sequence variation and patterns of substitution

Of the 1054 positions analyzed in the full (161-sequence) dataset, 729 sites were invariant and 229 were parsimony informative (including both ingroup and outgroup sequences). The condensed (99-sequence) dataset had less informative variation (760 invariant and 205 parsimony informative sites), but 42% of this difference was due to the exclusion of the *P. huachucanus* outgroup from the reduced dataset. As is generally expected for coding sequences, the vast majority of substitutions observed in our *cox1* sequences seem to be restricted to the degenerate third position (91% of pairwise differences). The high level of between site rate variability was also reflected in our various estimates of the gamma shape parameter using Modeltest and MrBayes, all of which suggested an alpha less than 1.0. The plots in Fig.2 also reveal a significant bias in the substitution rates for transitions and transversions, and there is strong evidence for transition saturation at the third codon position. However, a partitioned analysis reveals that it is only the more distant outgroup samples that show a marked decline in their transition/transversion ratio; thus, saturation is unlikely to significantly confound any of our ingroup comparisons.

### Phylogenetic results

A comparison of consensus trees from each of the six independent Bayesian analyses on this dataset revealed strong agreement among replicate runs, with identical topologies and similarly high support values for all major nodes. A 50% consensus phylogram from the sixth run, which provided the largest pool of quasi-independent replicate trees sampled at stationarity, is shown here with Bayesian posterior probability (BPP) values and average branch lengths estimated from 45,000 trees (Fig.3). Most major branches had strong support (i.e., BPP values ≥0.95), but we accepted major clades with BPP ≥0.85 as reasonably well supported. The focal species pair, *P. barbatus* and *P. rugosus*, were strongly supported (BPP=1.00) as a monophyletic ingroup relative to the *P. badius* and *P. bicolor* samples, although only *P. badius* was manually assigned to root the tree. Even after setting aside the established mtDNA introgression of several GCD lineages (J1 and H1/H2), the two putative species groups could not be considered monophyletic because of at least two other cases where nominal *P. rugosus* samples were recovered within the larger *P. barbatus* clade. To simplify the discussion of these inconsistencies, all samples were given a nominal mtDNA species tag according to the initial species bifurcation in the phylogeny (Table1). To facilitate various population genetic analyses on subdivision, divergence, and demographic expansion, major clades in the phylogeny were divided into a series of macro- and subgroups (see Methods). All samples from the previously characterized populations with GCD were restricted to just two monophyletic clades, in a pattern largely consistent with the phylogenies suggested by Anderson et al. (2006) and Schwander et al. (2007a). The fifteen J1 lineage samples were recovered in a single, moderately well-supported (BPP = 0.87) monophyletic clade within the *P. rugosus* species subtree (Fig.3). Twelve of the fifteen total J1 samples were contained within a strongly supported subclade (BPP = 1.00), which is more consistent with the well-supported J1 clade identified in Anderson et al. (2006). The remaining three J1 samples form a less well-supported (BPP = 0.78) sister group to the primary J1 subclade (Fig.3), and they were collected in a previously unsampled portion of the J1/J2 range at the southeastern portion of the Apache Highlands Ecoregion in Mexico.

The second GCD clade is larger and contains three lineages: J2, H1, and H2. As reported previously (Anderson et al. 2006; Schwander et al. 2007a), the known H1 and H2 lineage samples did not assort into reciprocally monophyletic mtDNA clades, but the combined H lineages clade was strongly supported as monophyletic and sister to the J2 lineage (Fig.3). Interestingly, the J2 lineage samples were not supported as monophyletic; rather, they were divided between two subclades that formed an unresolved multifurcation together with the combined H lineages clade (Fig.3).

In total, these clades include 5 newly discovered populations of J lineage *P. barbatus* and 24 new populations of H lineage *P. rugosus*. Taken together, these samples dramatically increase the inferred distribution of populations with GCD. Our results also suggest that the J2 lineage of *P. barbatus*, which is centered on southeastern Arizona, is more closely related to geographically distant populations of *P. barbatus* in southern Mexico than to the eastern group of ECD *P. barbatus* found in New Mexico and Texas. In addition to the MX2 sample included in Anderson et al. (2006), which is recovered here as a long terminal branch rooting the J2/H clade, our analyses recovered the J2/H clade as sister to a broadly distributed group of *P. barbatus* ranging throughout the southern Altiplano of Mexico (SWest Pbar, Fig.4). In contrast, the populations of ECD *P. barbatus* in the U.S. appear to be the northern extent of a broadly distributed eastern clade that extends south through the northeastern margins of the Chihuahuan Desert, and down the Gulf coast through the Mexican states of Tamaulipas and Veracruz (Fig.4). Both the SWest Pbar and the East Pbar clades contain a second well-supported bifurcation, which further splits them along a roughly north–south axis (Figs.3, 5).

This pattern is further informed by the geographic position of the macrogroup designated as Basal Pbar. The two clades in this group (Basal Pbar North and Basal Pbar South) are not supported as a monophyletic clade. However, the two pairs of samples were considered a meaningful assemblage because of their jointly narrow distribution along the western edge of the Sierra Madre Oriental, and because they are both relatively depauperate basal branches that may be an early divergence from the more broadly distributed clades in the *P. barbatus* mtDNA subtree. Notably, the two populations in the Basal Pbar North group were identified as members of the *P. rugosus* morphospecies (Pr445 and Pr451). In addition to the Basal Pbar North samples and the whole of the H lineage clade, one other sample with a *P. rugosus*-like morphology was recovered in the East Pbar 1 clade (Pr425). Moreover, the *cox1* sequence from Pr425 differed from that of the Pb419 sample by only one base pair, and the Pb419 sample also possessed a somewhat intermediate morphology. The relationship between geographical distributions and phylogenetic structure in *P. barbatus* is summarized in Fig.7.

The *P. rugosus* mtDNA species phylogeny was more straightforward, with seven nominal subgroups recovered in a progressively nested series of clades (Fig.3). The broadly distributed J1 and Prug 3 clades were recovered together as a monophyletic group, and they are progressively rooted by two other broadly distributed clades, designated Prug 2 and Prug 1. These three clades are rendered paraphyletic by the presence of the introgressed J1 lineage, which has a *P. barbatus*-like morphology, but they were nevertheless assembled into the nominal North Prug macrogroup because they represent the vast majority of the *P. rugosus* distribution, including all populations with a known ECD phenotype. The remaining three subgroups in South Prug are also a paraphyletic assemblage, but they were grouped together because they represent the more narrowly distributed basal clades for the species. The South Prug clades are especially interesting because they are distributed in three adjacent biogeographic regions, separated by well-studied vicariance barriers (the Sea of Cortes and the Sierra Madres Occidental). Thus, their positions and relative levels of divergence may provide some insight into the early patterns of dispersal and vicariance for the *P. rugosus* species.

The heuristic maximum-parsimony (MP) search, employed with the same condensed dataset used for all Bayesian analyses, identified 507,200 equally parsimonious trees of 909 steps. Despite this seemingly large number of trees, a consensus cladogram (not shown) revealed a well-resolved topology with 100% agreement for all major nodes. In contrast to the Bayesian phylogeny, this analysis placed the MX2 sample at a basal position within the J2 clade, rooted by the exceptionally diverged 2BAR and 7BAR haplotypes. This pattern is identical to that shown in Anderson et al. (2006). The strict consensus cladogram also reversed the positions of Prug 2 and Prug 3 relative to the Bayesian tree, making the south Sonoran Desert Prug 2 clade sister to the J1 lineage. Aside from these minor exceptions, the strict consensus cladogram recovered a nearly identical topology to that seen with our Bayesian analyses, including broad agreement on all other macro- and subgroup clades. The second MP analysis, conducted with rapid search parameters on the full (161-sequence) dataset, recovered an identical topology to the 99-sequence analysis described above. As this is a replicate analysis, it should not be taken as additional support for the topology in question, but it does suggest that the use of the reduced dataset did not adversely affect our analyses with the MP criterion.

We also constructed a 50% majority-rule consensus tree from the results of our MP bootstrap analysis, and because this tree was again largely consistent with the topology recovered from our Bayesian analyses, the bootstrap support (BSS) values were mapped onto the Bayesian phylogram (Fig.3). Unlike the initial MP analyses, the J2 and H clades were again recovered as monophyletic. However, the variability among bootstrap replicates was sufficient to collapse several of the shorter branches, which reduced both the Prug 3 and the H lineage clades into unresolved multifurcations, with their terminal branches sister to the J1/Prug 2 and J2 clades, respectively.

The results from our sensitivity analysis, which used multiple runs with either the NJ or the ME criterion, indicate that the topology is fairly stable to variations in our approach to both missing nucleotide sites and the exclusion of a large number of highly similar samples. The two criteria also recovered very similar topologies, except that ME consensus trees produced better resolution for the deeper relationships among major clades (discussed below). However, when comparing iterative runs within each criterion, only one major difference was observed, and only in NJ analyses. That is, the reduced (99-sequence) dataset recovered stronger bootstrap support than the full (161-sequence) alignment for the basal position of the Basal Pbar clade relative to the remainder of the *P. rugosus* subtree. Several other major clades showed a similar trend, with higher BSS in the reduced dataset consensus tree, but none varied by more than 5%.

Overall, the distance-based tree searches recovered similar subgroup clades to those described for the MP and Bayesian analyses. There were several notable exceptions however, similar to the variability observed in MP analyses. The largest difference was the formation of a seventh major clade in the *P. rugosus* subtree, composed of the longest branched samples in the Prug 1 clade (38, 39, and 43RUG) and the longest branched samples in the Prug 3 clade (30 and 32RUG). This collection of long branched samples from Anderson et al. (2006) was placed as sister group to the Basal Pbar clade. The only other major difference in the *P. rugosus* subtree was with the J1 group, which was split into two well-supported clades that were sister to Prug 2. The *P. barbatus* subtree showed a similar pattern of rearrangements, with three conspicuously long branched samples in the J2 clade (2, 6, & 7BAR), moved to a more basal position outside the SWest Pbar macrogroup. Also, the longest terminal branch in the SWest Pbar (Pb441) was recovered as a basal outgroup to the East Pbar macrogroup.

Most of the remaining clades received moderate to strong bootstrap support under both criteria, consistent with the BSS values seen with our MP analyses. However, both the NJ and the ME tree searches failed to achieve even 50% agreement among bootstrap replicates for most of the major nodes among well-supported clades. The 50% consensus trees were thus poorly resolved, with several broad multifurcations defining the relationships among most of the major clades for each species’ subtree. As observed with the MP bootstrap consensus tree, both the NJ and ME trees collapsed the H lineage clade into a broad multifurcation of terminal branches, which remained monophyletic within the broader J2/H clade.

### Divergence, population structure, and demographic history

Percentages of uncorrected pairwise sequence divergences are reported as averages within and between groups at three levels of phylogenetic and geographic inference. Species divergence (Table2) was assessed according to the mtDNA species bifurcation in the phylogeny. Estimates of mitochondrial sequence divergence among morphological species would be less informative because of the prevalence of horizontally transferred mtDNA between the sister species. Tables3 and 4 show average divergence among and within nominal macrogroups (Table3) and subgroups (Table4). Taken together, these data reveal several notable patterns which are consistent with the salient features of the phylogram shown in Fig.3. Average species level divergence is 6.6%, whereas the within-species averages were predictably lower (3.5% for *P. barbatus* haplotypes and 2.6% for *P. rugosus*). However, there was considerable variation in the level of divergence observed among major clades of *P. barbatus*, with values ranging from 2.1% to 5.6%. The largest of these values coincide with three (or four) basal splits which define the East Pbar clades, the combined SWest Pbar/J2 & H clade, and the narrowly distributed populations collected in the Basal Pbar group. By comparison, the divisions between J2 and H (2.1%), SWest Pbar 1 and SWest Pbar 2 (2.7%), and East Pbar 1 and East Pbar 2 (2.3%), appear to be much more recent.

**Table 2:**
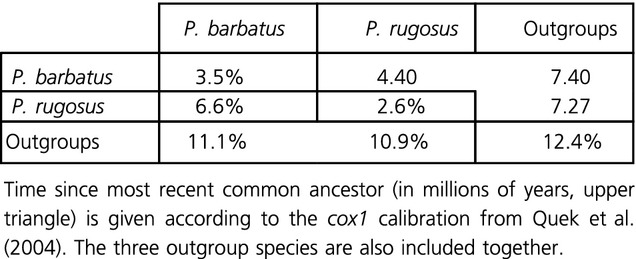
Average uncorrected pairwise distances within (center diagonal) and between (lower triangle) each mtDNA species subtree.

**Table 3:**
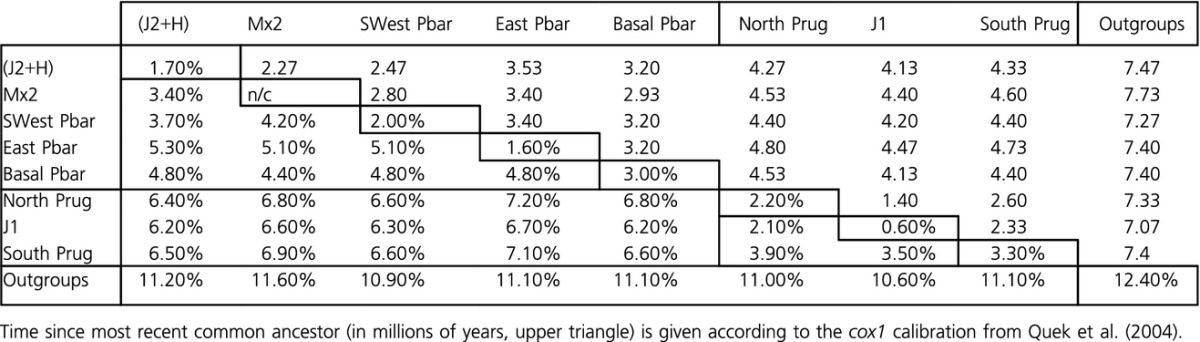
Average uncorrected pairwise distances within (center diagonal) and between (lower triangle) all nominal macrogroups as identified in the phylogeny (Fig.3).

**Table 4:**
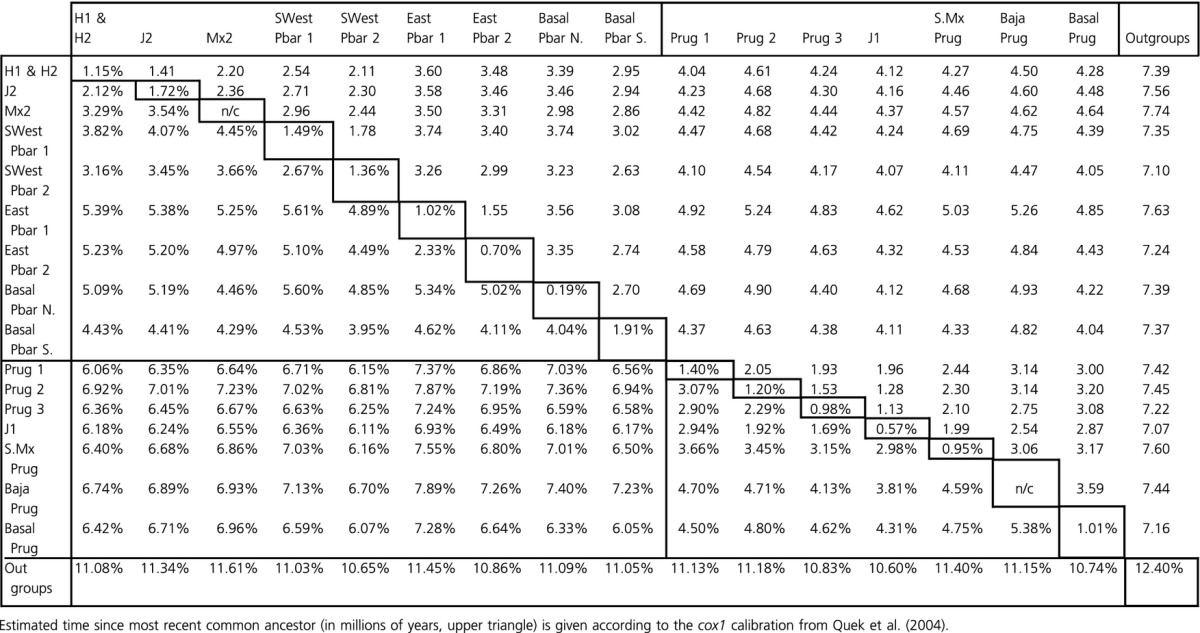
Average uncorrected pairwise distances within (center diagonal) and between (lower triangle) all nominal subgroups as identified in the phylogeny (Fig.3).

As mentioned, the *P. rugosus* subtree shows less average internal divergence, and this can be explained by two features of the data. First, the vast majority of ECD *P. rugosus* populations sampled (41/52) fall into the three North Prug clades (Prug 1-3), and the divisions between these clades appear more recent (2.3% to 3.1%) than the basal splits observed in *P. barbatus*. Second, the average distance between the two most basal subgroups in *P. rugosus* (Baja Prug and Basal Prug, 5.4%) is similar to the average distances among basal branches in *P. barbatus* (4.0% to 5.6%), but the Basal Prug and Baja Prug subgroups seem to be relatively narrowly distributed and are represented by just 5 total samples.

The results from our pairwise F_ST_s showed strong support for all nominal subgroups with >2 samples (i.e., all but Baja Prug, MX2, Basal Pbar North, and Basal Pbar South). The analyses were rerun without Baja Prug and MX2, and with a combined Basal Pbar subgroup, and all subgroups were strongly supported (P < 0.005; Tables S1 and S2).

The results from our AMOVAs supported a hypothesis of regionally nested population structure within mtDNA *P. barbatus* (i.e., differentiation between macrogroups and between subgroups), but only a single level of structure in *P. rugosus* (i.e., differentiation between subgroups but not between macrogroups). These results are shown in Table5, based on population structures that excluded singleton groups (Baja Prug and MX2), merged the two doubleton groups (Basal Pbar N. and S.) as a single subgroup within their own macrogroup, and merged J1 with the rest of Prug North so that macrogroup was monophyletic (thus, there were 4 macrogroups and 7 subgroups for mtDNA *P. barbatus*, and 2 macrogroups with 6 subgroups for mtDNA *P. rugosus*). To test the robustness of these results, the AMOVAs were repeated with several other hypothetical population structures consistent with the phylogeny for each mtDNA species (i.e., including the singleton and/or doubleton groups and treating J1 as a separate macrogroup; data not shown). Only one alternative population structure had an effect on the results (the inclusion of Baja Prug yielded a significant F_CT_ value for *P. rugosus*, see Discussion).

**Table 5:** Hierarchical analyses of molecular variance (AMOVAs) showing the partitioning of haplotype variance and tests for nested population structure in each mtDNA species tree (see Methods).

MtDNA species	Source of variation	df	Sum of squares	Variance components	% variation	F-statistics (*P*-value)
*P. barbatus*	Among macrogroups	3	820.796	11.74776	50.14	***F***_**CT**_ = 0.501 (0.00741)^*^
	Among subgroups within macrogroups	3	220.168	5.04698	21.54	***F***_**SC**_ = 0.432 (<0.0005)^*^
	Within subgroups	83	550.892	6.63725	28.33	***F***_**ST**_ = 0.717 (<0.0005)^*^
	Total	89	1591.856	23.43199		
*P. rugosus*	Among macrogroups	1	122.889	3.63329	21.42	*F*_CT_ = 0.214 (0.07065)
	Among subgroups within macrogroups	4	399.858	8.35439	49.24	***F***_**SC**_ = 0.627 (<0.0005)^*^
	Within subgroups	60	298.678	4.97797	29.34	***F***_**ST**_ = 0.707 (<0.0005)^*^
	Total	65	821.424	16.96565		

Significant values are marked in bold with an asterisk (P < 0.01). All nominal macrogroups and subgroups with >2 samples were included (thus, MX2 and Baja Prug were excluded, and Basal Pbar N. and S. are here combined), but J1 was merged with Prug North at the macrogroup level for better consistency with the phylogeny (Fig.3). F-statistics test for significant population substructure between macrogroups (*F*_CT_), between subgroups within their corresponding macrogroup (*F*_SC_), and between all subgroups regardless of nested macrogroup structure (*F*_ST_). Additional AMOVAs were run to assess the impact of alternative structure hypotheses on these results, but estimates of significance at each level were unaffected (see Results).

The results of our tests with *R*^2^, Tajima’s *D*, Fu’s *Fs*, and the mismatch distribution analysis provided mixed support for a hypothesis of recent population expansion in some of the subgroups (Table6). None of the Tajima’s *D* estimates were significantly different from 0, suggesting no deviations from neutrality as might be expected under a model of selection, bottlenecks, or expansion. In contrast, all of the mismatch distribution tests were nonsignificant, which indicates that the data for each subgroup were consistent with a model of recent population expansion. The two other tests, *R*^2^ and Fu’s *Fs*, produced a less uniform picture across subgroups. Two of the thirteen subgroups yielded significant values for *R*^2^ (Prug 3 and J1), and six of thirteen yielded significant values for Fu’s *Fs* (H lineages, SWest Pbar 1, East Pbar 1, East Pbar 2, Prug 3, and J1). For both *R*^2^ and *Fs*, significant values indicate deviations from neutrality consistent with a recent population expansion or selective sweep (Fu 1997; Ramos-Onsins and Rozas 2002).

**Table 6:** Five tests for recent population expansion in all nominal subgroups (as identified in Fig.3 and Methods, except Basal Pbar N. and S. are here combined, see Results).

Subgroup	*n*	*R*^2^ (*P*-value)	Tajima’s *D* (*P*-value)	Fu’s *Fs* (*P*-value)	*SSD* (*P*-value)	*RI* (*P*-value)
H1 & H2	33	0.08270 (0.149)	−0.7755 (0.227)	−7.6896 (0.013)^*^	0.0081 (0.3125)	0.0117 (0.438)
J2	17	0.15136 (0.735)	0.4348 (0.7237)	−1.2268 (0.2637)	0.0242 (0.4975)	0.0335 (0.447)
Mx2	1	–	–	–	–	–
SWest Pbar 1	11	0.14921 (0.457)	−0.0434 (0.5253)	−3.0567 (0.0487)^*^	0.0243 (0.476)	0.0298 (0.7405)
SWest Pbar 2	6	0.17467 (0.316)	−0.1696 (0.465)	−0.4599 (0.228)	0.0385 (0.8045)	0.0444 (0.9895)
East Pbar 1	9	0.12517 (0.0967)	−0.6864 (0.258)	−2.7712 (0.045)^*^	0.0447 (0.291)	0.1188 (0.155)
East Pbar 2	10	0.13936 (0.248)	−0.1982 (0.4663)	−4.5686 (0.0137)^*^	0.0189 (0.4175)	0.0365 (0.5985)
Basal Pbar	4	0.21349 (0.393)	0.9934 (0.8253)	1.6063 (0.492)	0.1699 (0.115)	0.4444 (0.455)
Prug 1	13	0.17325 (0.781)	0.7096 (0.807)	2.0364 (0.8307)	0.0518 (0.259)	0.0692 (0.266)
Prug 2	8	0.12842 (0.085)	−0.6146 (0.2927)	−2.0593 (0.0843)	0.0211 (0.697)	0.0485 (0.6745)
Prug 3	20	0.07867 (0.0187)^*^	−1.3038 (0.09)	−5.4379 (0.0197)^*^	0.0144 (0.2495)	0.0159 (0.565)
J1	15	0.09072 (0.0317)^*^	−1.1511 (0.1263)	−4.1004 (0.027)^*^	0.009 (0.8625)	0.0216 (0.8925)
S.Mx Prug	6	0.14188 (0.0917)	−0.4255 (0.381)	0.7842 (0.573)	0.0688 (0.5295)	0.1556 (0.488)
Baja Prug	1	–	–	–	–	–
Basal Prug	4	0.24124 (0.439)	−0.2278 (0.5753)	0.4093 (0.3393)	0.2323 (0.064)	0.5556 (0.4315)

Significant values are marked in bold with an asterisk (*P* < 0.05). For *D*, *Fs*, and *R*^2^, significance indicates a deviation from neutrality and support for a hypothesis of population expansion. For *SSD* and *RI*, significance indicates a deviation from the estimated model of demographic expansion based on mismatch analysis; thus, nonsignificant values indicate support for population expansion. *R*^2^ was calculated in DnaSP; all other statistics were calculated in Arlequin (see Methods).

## Discussion

his study has three primary goals: For the first time, we describe nearly the complete range of the *P. barbatus*/*P. rugosus* species complex with a molecular marker, identifying a series of genetically distinct phylogroups within the nominal morphospecies, and delineating the boundaries of the reproductively isolated J and H lineage pairs, which have been widely studied in the U.S.A. because of their association with a unique genetic caste determination (GCD) phenotype and evidence for historical hybrid introgression. Although we do not designate any of these phylogroups as formal subspecies, we hope this mtDNA framework and the recognition of such cryptic divisions will enable and encourage future researchers to evaluate their population-scale studies in the broader phylogeographic context. Such context is needed if we wish to assess whether a reported phenotype is likely to be representative of the species as a whole or a more narrowly distributed ecotype.

TSecond, we compare the phylogeographic and population genetic patterns within these species to the broader phylogeographic patterns identified for other taxa in these regions. This approach serves the dual purpose of adding data from an understudied taxon (social insects) to the phylogeographic literature, and identifying concordant patterns with better studied organisms in the literature should provide a hypothetical foundation for future research on these and other North American ant species (e.g., by providing estimates for age, historical barriers and vicariance, and possible subspecies divisions).

Third, by generating a more complete intraspecific phylogeny of *P. barbatus*, *P. rugosus*, J1, J2, and the H lineages, we can generate new insights on some of the published hypotheses about their hybrid ancestry and competitive viability.

### Reevaluating the *P. barbatus* and *P. rugosus* species distribution maps

Our efforts to sample and delineate the full range of both species, including especially the reproductively isolated J and H lineages with genetic caste determination (GCD), have revealed several key areas where previous distribution maps have failed to distinguish between the cryptic GCD lineages and the ancestral *P. rugosus*/*P. barbatus* species. This discrepancy has arisen because previous surveys were limited to morphological analysis when rendering species assignments, and it demonstrates the importance of using molecular markers to examine phylogeographic relationships within broadly distributed taxa. Chief among these corrections is the finding that approximately half the previously reported distribution for the *P. rugosus* morphospecies (i.e., the vast majority of areas east of the Sierra Madre Occidental and the Deming plains) appears to be exclusively inhabited by the hybrid H lineages.

In addition to the widespread J and H hybrid lineages, this study has identified two more clades in the *P. barbatus* mtDNA tree where one or more populations possess an incongruous morphology (*P. rugosus*-like, per Cole 1968; Pr445-G15 and Pr425-G17 in Fig.3). These two sets of putative hybrids are geographically and phylogenetically distinct, but they both occupy relatively narrow distributions near the center of the Mexican Altiplano. The Pr425 sample was assigned to East Pbar 1 (Fig.3) because its closest relatives are populations of *P. barbatus* in northern Mexico, Texas, and New Mexico (Fig.5). Pr425 is reminiscent of two ECD hybrid populations reported in Schwander et al. (2007a), which possessed a *P. rugosus* morphology but nuclear and mtDNA alleles consistent with admixture from *P. barbatus*. These two Schwander et al. (2007a) populations were found in Texas near the Rio Grande, and their geographic and phylogenetic positions suggest they are likely members of the East Pbar clade identified in the present study, but it is unclear whether these populations are the result of independent, local hybridization events or a more broadly distributed hybrid clade similar to the J and H lineages.

**Figure 6:**
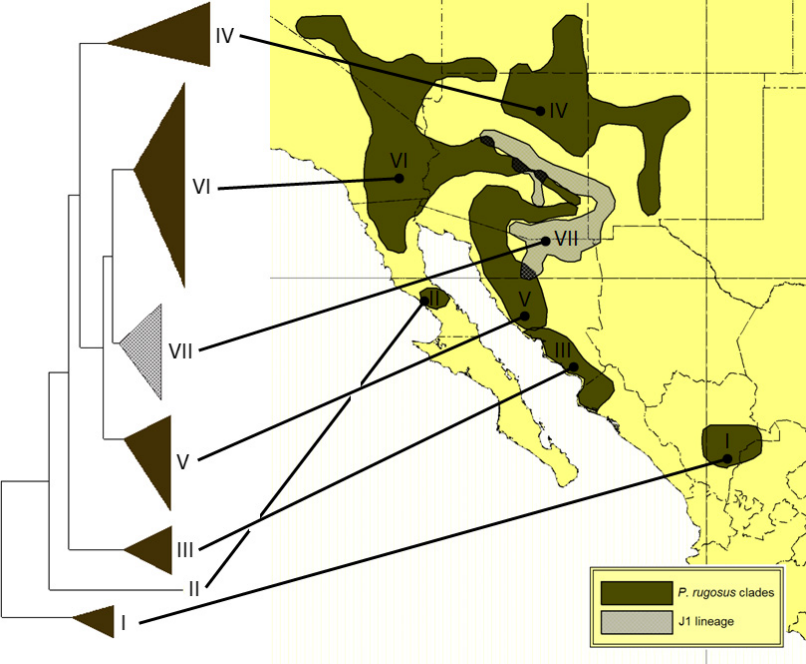
A simplified phylogram and regional map of the *Pogonomyrmex rugosus* subtree. Branches marked with Roman numerals in the tree (left) correspond to well-supported subgroups identified in the phylogeny (Fig.3). The distribution estimates (right) are based on population localities shown in the subgroups map (Fig.5). The J1 lineage (VII) is not a member of the *P. rugosus* morphospecies, but it is included here because of its hybrid-introgressed mitochondria.

**Figure 7:**
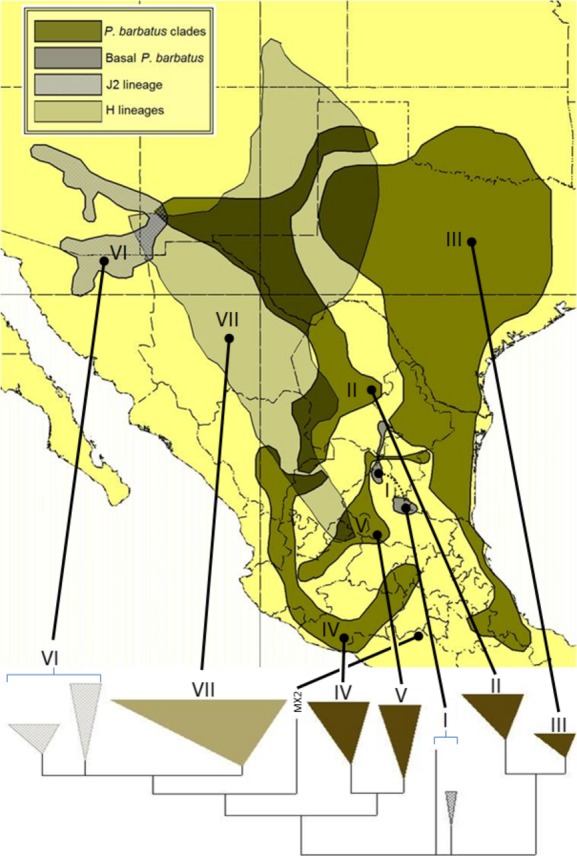
A simplified phylogram and regional map of the *Pogonomyrmex barbatus* subtree. Branches marked with Roman numerals in the tree (bottom) correspond to well-supported subgroups identified in the phylogeny (Fig.3). The distribution estimates (top) are based on population localities shown in the subgroups map (Fig.5). The H lineages (VII) are not members of the *P. barbatus* morphospecies, but they are included here because of their hybrid-introgressed mitochondria.

Additionally, Pr445-G15 occupies a basal position in the *P. barbatus* tree, which suggests that its incongruous morphology may be better explained by incomplete lineage sorting rather than hybridization. Regardless, these newly discovered hybrid groups combine with the vast distribution of hybrid H lineages to reveal a dramatically reduced distribution of mitochondrially defined *P. rugosus*, which primarily occupies the western and northern arid regions (Fig.6), and a correspondingly enlarged distribution of *P. barbatus*-derived mitotypes in the east (Fig.7).

In addition to these discrepancies resulting from cryptic mtDNA variation, our focused sampling in several areas has suggested two large regions where *P. barbatus* has previously been reported (from a few scattered collections), but now appears to be absent, and a number of previously unreported (as far as we aware) populations from both species at the southern limits of their range (see Appendix S1 for details). In general however, our findings on the distributions of the two morphospecies are largely consistent with the descriptions from Cole (1968) and Johnson (2000a).

As reported previously, we found extensive areas of overlap along regional contact zones between the *P. barbatus* and *P. rugosus* morphospecies (Cole 1968), and this regional overlap is likely facilitated by the tendency for the two species to segregate at local scales according to microhabitat differences in soil and moisture (Johnson 2000b). However, we also identified broad areas of apparent allopatry for each morphospecies, and if we recognize the J and H lineage pairs as cryptic species, then the true *P. rugosus* and *P. barbatus* distributions are largely allopatric.

### Evidence for regional structure and recent population expansions

In addition to the reproductively isolated J and H lineages, this study provides evidence for at least 12 more phylogroups with divergent mitotypes and geographically segregated distributions within each species (Figs.5). The results from our AMOVAs (Table5) and pairwise F_ST_s (Tables S1 and S2) strongly support the subgroups identified in the phylogeny (Fig.3). In mitochondrial *P. barbatus*, the results from our hierarchical AMOVA indicated strong support for a hypothesis of nested regional population structure (i.e., subgroups within macrogroups). This result supports the hypothesis for a broad east–west division within *P. barbatus*, and it recognizes the J2/H clades and Basal Pbar populations as possessing distinct mitochondrial DNA diversity.

In mitochondrial *P. rugosus*, the AMOVA indicated strong support for population structure between subgroups, but the test for regional structure between North Prug and South Prug was not significant (*P* = 0.07065, Table5). In fact, our hypothesized population structure (six subgroups, two in one group and four in another) has a technical *P*-value limit of 0.0667 because of the permutation scheme employed for the test (Fitzpatrick 2009). Moreover, when we included the Baja Prug subgroup in the structure hypothesis, the regional test was significant (*F*_CT_ = 0.19227, *P*-value = 0.03854). However, as the Baja Prug sample is represented by only a single sample, and as a significant *F*_CT_ does not significantly change our interpretation of the regional structure in *P. rugosus* (as South Prug was recognized a priori as a paraphyletic assemblage), we are reporting the more conservative estimates in Table5. More generally, it should be noted that these AMOVAs and pairwise *F*_ST_s are testing groups defined a posteriori from the well-supported clades in the phylogeny. To properly validate these groups and the regional hypotheses they represent, future studies will need to test them under an a priori framework with additional independent markers. It is likely that gene flow still exists between many of the non-GCD subgroups identified within each morphospecies, and the patterns of apparent geographic structure in their mitochondrial DNA are the result of historical processes or an artifact of low sample size and isolation by distance.

As we are expecting many of these subgroups to have undergone significant range contractions during the Pleistocene, we evaluated each subgroup with a series of tests sensitive to the genetic signals created by recent increases in population size (Table6). The results from these tests produced a mixed picture for the individual subgroups, with some tests showing evidence for population expansion where others did not. However, some of these conflicting results may be explained by known limitations of the tests. Specifically, Tajima’s *D* (Tajima 1996) is known to be less powerful for detecting population expansion than either Fu’s *Fs* or *R*^2^ (Fu 1997; Ramos-Onsins and Rozas 2002), so it is not surprising that the subgroups with significant estimates for Fu’s *Fs* and/or *R*^2^ have negative but not necessarily significant values for Tajima’s *D*. We were surprised, however, that all 13 subgroups evaluated with mismatch distributions were found to have haplotype data consistent with a model of recent population expansion (see Table6 and Results). We are unaware of any robust comparisons on the performance of mismatch distributions versus the tests of neutrality (*D*, *Fs*¸ and *R*^2^), but we are less confident in the results from our mismatch distribution analyses because their implementation in Arlequin 3.5 (Excoffier et al. 2005) only allows for a null hypothesis of population expansion.

Thus, we will rely primarily on our results from testing with *Fs* and *R*^2^ as evidence for recent population expansion or selective sweeps. Six of the thirteen subgroups produced significantly negative estimates for Fu’s *Fs*, but only two of these six groups (Prug 3 and J1) were also significant for *R*^2^ (Table6). This is surprising because *R*^2^ has been suggested as potentially more powerful than Fu’s *Fs* in groups with low sample size (Ramos-Onsins and Rozas 2002). Additionally, studies have shown that the presence of population substructure within a group can create a signal similar to that expected from a recent increase in population size, and a broad, low-density sampling strategy like ours can exacerbate this phenomenon (Hammer et al. 2003). This may be especially pertinent for interpreting the statistically significant Fu’s *Fs* estimate in the nominal H lineages, where we have an a priori expectation for structure between the two dependent lineages. Clearly, these demographic estimates will need to be confirmed with additional sampling in future studies.

### Inferring caste determination phenotype for newly sampled populations

As noted in the methods, the current study did not test individual populations to confirm their caste determination phenotype. However, we used previously assayed samples with a known ECD or GCD phenotype, and a simple parsimony model, to reconstruct the probable caste determination phenotype for ancestral nodes in our tree (see Methods and Fig. S1). As all known GCD haplotypes were recovered in just two monophyletic clades that contained no known ECD samples, and as the same was true for all known ECD samples, the ancestral state reconstruction was very straightforward. Only a single ancestral node (the ancestor of MX2 and the J2/H clade) was ambiguous, with equal evidence for either GCD or ECD (Fig.3). After identifying the likely phenotype of the ancestral nodes, we make the parsimonious inference that all samples with an unknown phenotype are likely to share the phenotype of their ancestral node (assignments listed in Table1) and, by extension, the phenotype of their most closely related living relatives.

However, it should be noted that our limited knowledge of the origins and genetic mechanisms of the GCD system necessarily means that we cannot definitively assign the ECD or GCD phenotype to any particular colony that has not been individually assayed. Rather, our approach here is focused on the phylogeography of the discrete mitochondrial lineages. It is possible that some of the H lineage populations have a different phenotype than their closely related brethren in the north, and it is also possible that some of the inferred ECD populations possess GCD. The SWest Pbar clade has only one distinct haplotype (two samples) with a known caste determination phenotype, so its inferred ECD phenotype may be especially tenuous.

Moreover, at least one study has found evidence for a partial- or incipient-GCD phenotype that appears distinct from the “strict GCD” described for the J and H lineages (Schwander and Keller 2008). This phenotype was described in a population of *P. rugosus* in Arizona, and they used somewhat different methods than had been employed for detecting dependent lineage pairs and caste determination phenotypes in previous studies (e.g., Helms Cahan and Keller 2003; Anderson et al. 2006, 2011; Schwander et al. 2007a). Unfortunately, no phylogenetic or gene flow data were provided for the colonies sampled, so it is unclear how this observation may relate to the broader J and H lineages or the presumed ECD lineages of *P. rugosus*. The results from Schwander and Keller (2008) illustrate two points: First, a definitive map of caste determination phenotypes will require much more detailed analyses, perhaps across every single population in the species complex. Second, our ability to extrapolate from this and the many other population-level studies depends on incorporating each newly described population and phenotype into a phylogeographic framework, so we can create a more cohesive picture of these patterns of cryptic variation.

### Evidence for a complex phylogeographic history

Our phylogenetic and population genetic analyses suggest a complex history that includes ancient intraspecific vicariance, fragmentation, hybridization, and recent expansion or recolonization of lost habitats in both species. Based on the concordance between our data and patterns of phylogeographic structure identified in studies of similarly distributed taxa, we hypothesize that most of the temporal and spatial complexity observed among these clades can be attributed to a combination of climate cycling in the Pleistocene and several major physiographic transformations in the early Pliocene and mid- to late Miocene.

As predicted, our analyses revealed a recurring pattern of broad east–west division among the most basal nodes of each species, corresponding to the four major north–south arid-land corridors of Mexico and the southwestern U.S.A. (i.e., Baja Peninsula, Sonoran–Sinaloan coastal province, Chihuahuan Desert/Mexican Altiplano, Gulf Coast/Tamaulipan Plain). A large number of phylogeographic studies point to these four corridors as areas of endemism and early divergence within arid-adapted species complexes (the first three corridors are reviewed in Riddle and Hafner 2006a; and the Gulf coastal plains are discussed in Riddle and Honeycutt 1990; Castoe et al. 2007; and Mulcahy 2008, among others). The southern half of the Mexican Altiplano is home to multiple basal nodes from the two species, including several apparent relict groups, which suggests the region may be a good candidate for further investigation into the early diversification and speciation of lineages in the *P. barbatus*/*P. rugosus* species complex.

The cryptic fragmentation among seemingly contiguous distributions of northern *P. rugosus* and eastern and southern *P. barbatus* suggests that even the youngest of these clades predate the modern day Holocene, an interglacial period that began approximately 11,000 years ago (Van Devender 2000). During the last glacial maximum (LGM), a combination of forest expansions and pluvial lakes restricted desert communities throughout most of the Basin and Range province and on the Colorado Plateau (Spaulding et al. 1983; Betancourt 1990; Thompson et al. 1993). Only a few desert-like refugia have been identified in the midden record (e.g., Death Valley, the Lower Colorado River Valley, and somewhere in Sonora Mexico, Betancourt 1990), but there is also evidence for one or more desert refugia in the Chihuahuan Desert (e.g., the Bolsón de Mapimí is supported by Morafka 1977; Elias et al. 1995; Orange et al. 1999; Riddle and Hafner 2006a,b; Castoe et al. 2007; but see Van Devender and Burgess 1985; and a second refugium has been proposed north of the Rio Grande river, Smith and Farrell 2005). Furthermore, it is unclear how the patterns from the last glacial–interglacial cycle relate to ecological shifts in the earlier Pleistocene and Pliocene, which were subject to somewhat different climate conditions and which may have been influenced by Plio-Pleistocene uplift in the Sierra Nevada range (Betancourt 1990).

We hypothesize that the complex reflexive patterns observed in the northern *P. rugosus* (Fig.6) are probably the result of successive climate shifts that broke off peripheral portions of the ancestral distribution in the south (S.Mx Prug), then in the north (Prug 1), and then finally breaking apart the youngest clades in the center (Prug 2 and Prug 3). These reflexive or nested histories seem to be a common feature of finer scale phylogeographic analyses in the Sonoran and Mojave deserts, and often with a basal clade in southern Sonora (Douglas et al. 2006; Leaché and Mulcahy 2007; Leavitt et al. 2007; Mulcahy 2008). Consistent with the division between Prug 2 and Prug 3, there is abundant evidence for Pleistocene isolation between western/Mojave/LCRV and eastern/Sonoran clades (e.g., Riddle 1995; Jaeger et al. 2005; Douglas et al. 2006; Castoe et al. 2007; Leaché and Mulcahy 2007; Leavitt et al. 2007; McGuire et al. 2007; Mulcahy 2008; Jezkova et al. 2009). There is also evidence for recent population expansion in the Mojave/LCRV clade (Prug 3), as indicated by the large geographic distances between similar haplotypes and the statistically significant estimates for Fu’s *Fs* and *R*^2^ (Figs.3, 5, Table6). In contrast, the Prug 2 clade (which appears to occupy the Sonoran Desert, south of the Gila River) produced Fu’s *Fs* and *R*^2^ estimates that were not quite significant, suggesting that it may not have experienced a major expansion in recent Pleistocene/Holocene time (Table6).

Many of our phylogeographic hypotheses could be addressed in part by assigning accurate ages to major nodes in our phylogenetic tree. However, without the aid of a calibrated molecular clock and a detailed fossil record, our inferences for this study are primarily limited to relative comparisons within these two species. If we assume roughly clock-like sequence divergence for our samples, then we can infer that the oldest divisions in *P. rugosus* and *P. barbatus* are a little more than two times the age of the most recent divisions (e.g., ≈2.3% between Prug 2 and Prug 3 or between East Pbar 1 and East Pbar 2). If isolation between these youngest clades follows the predominant pattern of late Pliocene to mid-Pleistocene (1-3 mya) fragmentation indicated for major vertebrate clades in the Mojave and Sonoran deserts (Riddle 1995; Douglas et al. 2006; Riddle and Hafner 2006b; Leaché and Mulcahy 2007; Leavitt et al. 2007) and between interior and Gulf Coast clades in snakes (Castoe et al. 2007; Mulcahy 2008), then the more basal divisions are likely to have diverged around the Miocene–Pliocene transition 3–6 mya. This rough timeline coincides with estimates of the opening of the Sea of Cortes (Riddle et al. 2000a; but see Crews and Hedin 2006), and some estimates of early divergence across the Sierra Madre Occidental (Riddle and Hafner 2006b).

It is also possible, although imprecise, to evaluate this timeline by comparing our calculations of average between-clade divergence (Tables4) with estimates of *cox1* substitution rates in other organisms. Quek et al. generated such a calibration for their *Crematogaster* ant phylogeny by averaging *cox1* rates from several arthropod studies with what they called “tenable calibrations” for major nodes (2004). They found that rates were generally conserved, even among highly diverged taxa, and the three insect groups in their analysis converged around the overall average of 1.5% uncorrected p-distance between clades, per million years (Quek et al. 2004). This calibration suggested a timeline for *Crematogaster* divergence in South-East Asia that was consistent with biogeographic events independently inferred from plant fossils (Quek et al. 2004), and this rate was also supported by an independent estimate of *cox1* rates in a phylogeographic study of leafcutter ant evolution in South America (Solomon et al. 2008). Applying this rate to our data suggests a mid-Pleistocene divergence for the youngest clades (e.g., 1.1 mya for J1 and Prug 3) and an early Pliocene divergence for the oldest (e.g., 4.4 mya for the average between *P. barbatus* and *P. rugosus*). Although these estimates argue for a somewhat shallower timeline for *P. rugosus*, they are still consistent with the general model of late Miocene to Pliocene vicariance between – and Pleistocene-aged fragmentation within – major regional deserts, as described above. Nevertheless, we must caution the reader to view this timeline as highly tentative because it is based on data from only a single mitochondrial gene, the evidence available is indirect and hinges on the accuracy and applicability of patterns reported for other organisms. Moreover, our calculations of average pairwise divergence between clades represent an incomplete picture of intraspecific coalescence, so they may under- or overestimate the actual time since population divergence. It is likely that the inclusion of additional gene sequences and the use of an internally derived molecular clock will alter these results.

The division between the J and H lineages resembles a common arid-species pattern of east–west vicariance across the CFB in Pliocene–Pleistocene time (e.g., Riddle et al. 2000b; Devitt 2006; Riddle and Hafner 2006b; Castoe et al. 2007; Leaché and Mulcahy 2007), but the significance of this congruence is unclear. In other groups, Pleistocene divergence across the CFB can plausibly be attributed to evolution in allopatry after the formation of glacial woodland barriers indicated by midden fossils (Betancourt 1990), but it has been suggested that the divergence between the J2 and H lineages may have occurred in sympatry through hybrid speciation processes (Schwander et al. 2008).

The phylogeographic patterns for both *P. rugosus* and *P. barbatus* are covered in more detail in Appendix S2.

### Recognizing the J and H lineages as distinct biogeographic entities

The wide-ranging phylogeographic reconstructions in this study provide several key insights into the origins and evolution of the J- and H-dependent lineages pairs that display the GCD phenotype. Broader geographic sampling has simultaneously allowed us to better delineate the biogeographic and phylogenetic boundaries of the J and H lineage pairs, confirming that both lineage pairs represent independent evolutionary units via their exclusive physical distributions and cladistic monophyly. Although they show evidence of historical hybridization (Helms Cahan and Keller 2003; Anderson et al. 2006), several studies have confirmed that these lineages are an evolutionarily stable group with reproductive isolation from their nominal parental species (Anderson et al. 2006; Schwander et al. 2007a). However, previous studies have lacked the breadth of population sampling necessary to represent the dependent lineages and their nominal parental species in a regional biogeographic context. Indeed, all previous studies have centered on biogeographic intersections, particularly the Cochise Filter Barrier area where the Sonoran and Chihuahuan deserts meet, and thus, the J and H lineages have largely appeared as distributionally intermingled with their putative parental species, *P. rugosus* and *P. barbatus*. Although there is significant distributional overlap in the Cochise Filter Barrier area and throughout western Texas, we suggest that this depiction may be an artifact of sampling along regional ecotones and physiographic intersections, where Holocene climatic shifts are likely to have created new contact among previously isolated biogeographic provinces.

In contrast, the delineation of the J and H lineages in their respective, and at least partially discrete, distributions should advance their recognition as evolutionary independent units. The recognition of the J lineages as the dominant clade in the Apache Highlands Ecoregion (Anderson et al. 2011) more accurately ties that group to its unique ecological environment. Similarly, the more complete depiction of the surprisingly broad H lineages, which seem largely dominant throughout the Chihuahuan Desert, indicates that this group may have a unique ecological niche that is distinct from ECD *P. rugosus*, and it may have an advantage over its arid-adapted congeners.

Moreover, the use of broader, and sometimes denser, sampling has allowed us to better identify the geographic and phylogenetic positions of the putative parents for the J and H lineages. It has previously, and widely, been assumed that the ECD *P. barbatus* in New Mexico and Texas (termed East Pbar in this study) were the likely parents of the hybrid J and H lineages, which were first described near the Arizona and New Mexico border (Julian et al. 2002; Volny and Gordon 2002; Helms Cahan and Keller 2003). As such, populations from the East Pbar clades were used to investigate patterns of admixture and hybrid introgression among the J and H lineages (Helms Cahan and Keller 2003; Schwander et al. 2007a), and those studies suggested that the East Pbar have made major nuclear DNA contributions to both the J1 and the H lineages. However, the mtDNA analyses in this study clearly favor populations from south-central Mexico, including the MX2 sample and the broadly distributed SWest Pbar clades, as more closely related to the J2 and H lineages.

Because the J1 lineage has an introgressed mitochondria, it is impossible to determine its origin among *P. baratus* via mtDNA phylogenetics alone. However, J1’s phylogenetic pattern can be strongly linked to the J2 lineage by several indirect lines of evidence. First, the current distribution and life history of the J1 lineage seem immutably linked to J2, which leads us to presuppose a shared origin for the two J lineages. Additionally, the mitochondrial lineage of J1 appears to be derived from one of the western clades of *P. rugosus* in either the Sonoran or Mojave Desert. These desert *P. rugosus* clades occupy areas adjacent to the current distribution of J2 (and J1) in the Apache highlands, and all other putative *P. barbatus* parents for J1 are much further removed in New Mexico, Texas, or south-central Mexico (Fig.2). Thus, in terms of phylogeography and life history, the J2 lineage seems to be the most parsimonious source for the J1 lineage’s *P. barbatus* ancestry.

Unfortunately, this study did not recover any additional populations that were closely related to the MX2 sample, and we lack the nuclear data necessary to repeat the admixture analyses conducted in previous studies. Nevertheless, it seems clear that future studies on the hybrid character and putative origins of the GCD lineages would benefit from including these southern Mexico clades in their analyses.

### Origin and evolution of dependent lineage pairs with GCD

As defined in this study via mtDNA sequences, the H lineages can be traced to a single origin (i.e., they form a monophyletic clade), which means that the hybrid introgression that established the clade occurred as an effectively discrete event (i.e., either the introgression occurred in a narrow geographic space or the lineages were bottlenecked sometime after). This is consistent with the results from previous studies (Anderson et al. 2006; Schwander et al. 2007a), and it logically indicates that any populations that carry this hybrid signature must be the result of proliferation and expansion from this effectively discrete introgression event. If this presupposed radiation is relatively recent, then we should be able to detect this proliferation with our tests for recent population expansion. This hypothesis is partially supported by the statistically significant Fu’s *Fs* estimate in this subgroup, though not with *R*^2^ (Table6). If this pattern is supported in future studies, it may indicate the rapid expansion of the H lineages in response to shifts in climate and habitat availability in the late Pleistocene or Holocene. However, the exceptional breadth of the H lineage distribution may also indicate a competitive advantage for the clade, perhaps as a result of their hybrid ancestry or some as-yet unidentified benefit of the GCD phenotype.

As with the H lineages, the hybrid-introgressed mtDNA in the J lineage seems to form a monophyletic clade, which necessarily indicates that it has radiated out from an effectively discrete hybridization event. In J1, we found evidence for recent population expansion with both Fu’s *Fs* and *R*^2^ (Table6), which suggests that, like the H lineages, it probably expanded its distribution in the late Pleistocene or Holocene. However, the J2 clade does not share this signal for recent population expansion, and similar to Anderson et al. (2006), we found that the J2 clade contained far more internal genetic variation than either J1 or the combined H lineages (Table4). Despite sampling a similar number of populations across the same geographic range (J2 N = 17, J1 N = 15), the J2 clade’s internal p-distance estimate (1.72%) is approximately three times that of the J1 clade (0.57%).

These conflicting genetic patterns between J1 and J2 are surprising because the two lineages are always found in sympatry (Anderson et al. 2006, 2011; Schwander et al. 2007a), and our conceptual understanding of the GCD system necessitates that the two lineages expand and contract their distributions in concert because neither can survive without the other. However, studies have shown that J2 is actually more numerous in most populations (Anderson et al. 2011), and J1 colonies produce fewer female offspring on average (Anderson et al. 2009). These two factors could have a significant impact on the effective population size of J1 relative to J2, but we would not expect the approximately threefold difference observed in their internal p-distances. Thus, we conclude that much of the mtDNA variation in J2 precedes its obligate mutualism with J1. This conclusion does not definitively address the origins of the GCD phenotype, but it suggests that, in the time before its contact and co-evolution with the J1 mtDNA lineage, the J2 lineage must have occupied a relatively stable distribution throughout much of the Pleistocene, long before its contact with the J1 mtDNA lineage.

One model for the origin of GCD suggests that it may have evolved in the ancestral J2 clade first and then subsequently introgressed into the J1 and H lineages (Anderson et al. 2006). However, if the GCD phenotype, and its commensurate dependent lineage pairs, was already distributed throughout the J2 clade’s geographic distribution, then it is difficult to explain the rapid radiation of the newly formed J1 mtDNA clade through the populations of this established system. On the other hand, if we hypothesize that the introgression that created the new J1 mtDNA clade is also tied to the generation of some sort of egoistic gene complex (as hypothesized for the GCD phenotype; Anderson et al. 2006), then it is easier to imagine its rapid expansion throughout the established range of the J2 clade. Under this egoistic gene model, the ancestral J2 populations would presumably have undergone some initial process of genetic sorting when they contacted the GCD mechanism vectored through J1, but it is conceivable that these perturbations would have left the J2 clade’s mtDNA, and thus its deeper demographic history, intact. Interestingly, this model of egoistic gene invasion generates some testable predictions: Specifically, it hypothesizes a genetic reshuffling in J2 that should, at least in part, be idiosyncratic for each J2 population. As this hypothetical J1 ancestor was likely to be invading with a fresh supply of introgressed loci from *P. rugosus*, each of these hypothetical J2 ancestral populations might have received their own unique combination of *P. rugosus*-derived chromosomal elements.

## Supporting Information

**Figure S1.** 99-sequence phylogeny (topology identical to Fig. 3) depicting ancestral state reconstructions for the caste determination phenotype (see Methods). **Table S1.** Significance (*P*-values) from 2024 bootstrap permutations for all pairwise *F*_ST_ comparisons among the *P. barbatus* subgroups as calculated in Arlequin (see Methods). **Table S2.** Significance (*P*-values) from 2024 bootstrap permutations for all pairwise *F*_ST_ comparisons among the *P. rugosus* subgroups as calculated in Arlequin (see Methods). **Table S3.** Accession numbers. **Appendix S1.** Summary of discrepancies and proposed amendments regarding the distribution of *P. barbatus* and *P. rugosus* morphospecies. **Appendix S2.** Detailed analysis of *P. rugosus* and *P. barbatus* phylogeography.
