# Supplementary material for: Phylogeography of Pogonomyrmex barbatus and P. rugosus harvester ants with genetic and environmental caste determination
Source: Ecol Evol. 2015 Jun 25;5(14):2798–826. doi: 10.1002/ece3.1507 (PMC4541987; doi:10.1002/ece3.1507)
Supplement: Supplementary file 1 [file ece30005-2798-sd1.docx]

Supplemental material:


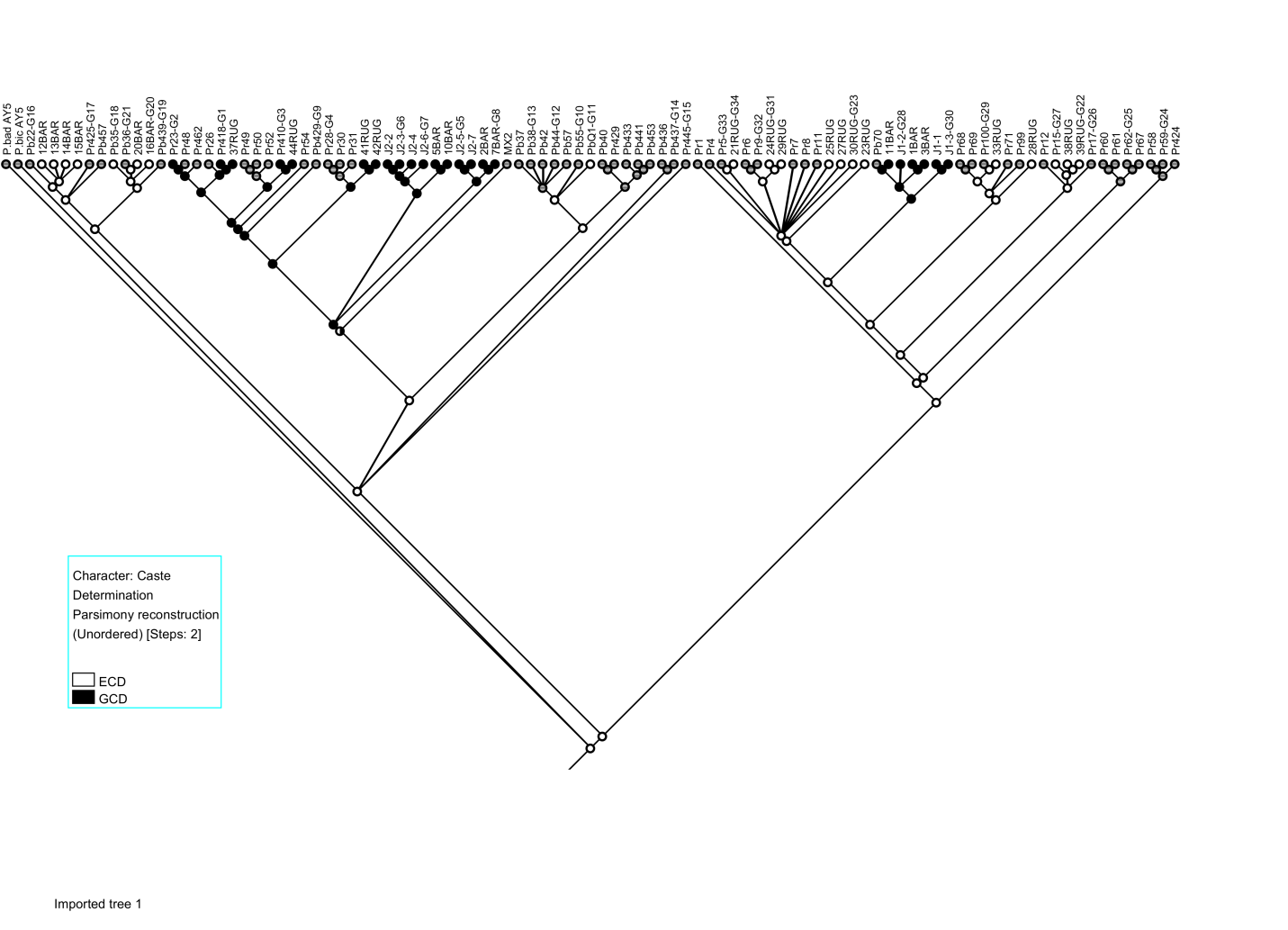


Supplemental Figure S1: 99-sequence phylogeny (topology identical to Fig. 3) depicting ancestral state reconstructions for the caste determination phenotype (see Methods). Leaves include 43 in-group samples with a known GCD (black filled circles) or ECD (white, unfilled circles) phenotype, plus 54 in-group samples with an unknown (grey filled circles) caste determination phenotype. Ancestral phenotype states were reconstructed based on a simple parsimony model, and these are depicted on internal nodes with the same color scheme as leaves. One node (connecting MX2 to the J2 and H clades) is ambiguous, with equal probability for either GCD or ECD, and is therefore depicted with a half-filled circle.

Supplemental Table S1: Significance (p-values) from 2024 bootstrap permutations for all pairwise F_ST_ comparisons among the *P. barbatus* subgroups as calculated in Arlequin (see Methods). Basal Pbar N. and S. are shown here as a single group, and the lone MX2 sample was excluded.

|  | EastPbar1 | EastPbar2 | H | J2 | SWestPbar1 | SWestPbar2 | BasalPbar |
| --- | --- | --- | --- | --- | --- | --- | --- |
| EastPbar1 | - |  |  |  |  |  |  |
| EastPbar2 | 0.00000+-0.0000 | - |  |  |  |  |  |
| H | 0.00000+-0.0000 | 0.00000+-0.0000 | - |  |  |  |  |
| J2 | 0.00000+-0.0000 | 0.00000+-0.0000 | 0.00000+-0.0000 | - |  |  |  |
| SWestPbar1 | 0.00000+-0.0000 | 0.00000+-0.0000 | 0.00000+-0.0000 | 0.00000+-0.0000 | - |  |  |
| SWestPbar2 | 0.00000+-0.0000 | 0.00000+-0.0000 | 0.00000+-0.0000 | 0.00000+-0.0000 | 0.00000+-0.0000 | - |  |
| BasalPbar | 0.00247+-0.0011 | 0.00099+-0.0007 | 0.00000+-0.0000 | 0.00000+-0.0000 | 0.00049+-0.0005 | 0.00494+-0.0017 | - |

Supplemental Table S2: Significance (p-values) from 2024 bootstrap permutations for all pairwise F_ST_ comparisons among the *P. rugosus* subgroups as calculated in Arlequin (see Methods). The lone Baja Prug sample was excluded.

|  | BasalPrug | SMxPrug | J1 | Prug1 | Prug2 | Prug3 |
| --- | --- | --- | --- | --- | --- | --- |
| BasalPrug | - |  |  |  |  |  |
| SMxPrug | 0.00494+-0.0017 | - |  |  |  |  |
| J1 | 0.00049+-0.0005 | 0.00000+-0.0000 | - |  |  |  |
| Prug1 | 0.00049+-0.0005 | 0.00000+-0.0000 | 0.00000+-0.0000 | - |  |  |
| Prug2 | 0.00148+-0.0008 | 0.00000+-0.0000 | 0.00000+-0.0000 | 0.00000+-0.0000 | - |  |
| Prug3 | 0.00000+-0.0000 | 0.00000+-0.0000 | 0.00000+-0.0000 | 0.00000+-0.0000 | 0.00000+-0.0000 | - |

>>>>

Supplemental Appendix S1: Summary of discrepancies and proposed amendments regarding the distribution of *P. barbatus* and *P. rugosus* morphospecies

This study includes a number of previously unreported (as far as we are aware) populations of *P. rugosus* and *P. barbatus* in the southern limits of their range, extending their known range in some areas of central Mexico. Conversely, our extensive sampling efforts in several areas (see Methods for details) also suggest that previous reports of *P. barbatus* in the central regions of the Sonoran and Chihuahuan deserts may be in error. Rather, these areas seem to be completely dominated by populations of *P. rugosus* (in Sonora) and H lineage hybrids (in Chihuahua), or other *Pogonomyrmex* species. Investigating the absence of a species from a region is a complicated endeavor because there is always the possibility that we simply failed to locate the reported populations. In general however, our depictions of morphospecies distributions in this study are highly consistent with the excellent surveys from Cole (1968), wherein he notes that *P. barbatus* is “…largely absent from northwestern Mexico, where it is replaced by *rugosus*.” Cole’s map of *P. barbatus* in Sonora and Arizona, which we now know as the cryptically diverged J lineages, also closely resembles our delineation of the southern and western boundaries of that group (Fig. 4).

The most significant contradiction between our distribution maps and previous reports on the morphospecies comes from Johnson (2000). That study includes reports of putative *P. barbatus* populations scattered through the southern thornscrub regions of Sonora and northern Sinaloa (see Fig. 6 in Johnson 2000a). We were unable to confirm the presence of *P. barbatus* in these areas, but we did encounter several populations of *P. rugosus* with a uniquely reddish-orange coloration on the head that initially led us to classify them as *P. barbatus* (Pr60 and Pr62 in Table 1). A more thorough morphological analysis (based on patterns of cephalic and promesonotal rugae as described in Cole 1968), led us to the conclusion that they were in fact members of *P. rugosus*. Our mtDNA analyses also indicated that they were *P. rugosus*. The two uniquely colored populations possessed somewhat disparate haplotypes, indicating that they were not themselves closely linked, but they were closely related to other populations of *P. rugosus* in the region with more typical coloration (Fig. 3). These two populations occurred along the Río Fuerte in Sinaloa, near one of the *P. barbatus* localities reported in Johnson (2000). At present, we can only speculate on the nature of this contradiction, but it may be that some or all of the reported instances of *P. barbatus* in this region should be reclassified as *P. rugosus* with a somewhat aberrant morphology. Alternatively, if the populations reported in Johnson (2000) are indeed derived from *P. barbatus*, then they may represent a southern extension of the J lineages or a relict population of the ancestral *P. barbatus* that has been isolated from its sister groups to the east. Either possibility suggests that these populations may be highly informative and that this discrepancy merits further investigation.

Overall, our observations in the field and our phylogeographic analyses argue for a more discrete representation of the species/lineages and their distributions. Although there are extensive areas of overlap along regional contact zones between *P. barbatus* and *P. rugosus* (Cole 1968), and this regional overlap is likely facilitated by their tendency to segregate at local scales according to microhabitat differences in soil and moisture (Johnson 2000b). An exhaustive accounting of all populations at the local scale is clearly beyond the scope of this study, and the authors cannot claim to have sampled or observed every extant population of *P. barbatus* and *P. rugosus*. Moreover, this study did not cover some areas of presumed allopatry where historical sampling has been robust (e.g., the northeastern limits of *P. barbatus* in Texas, Oklahoma, Louisiana, and Arkansas), and thus our distributional maps (Figs. 6 and 7) do not extend into these areas. However, we focused our sampling and field surveys along regional contact zones, and we endeavored to locate possible pocket populations of the subordinate species in regions of its apparent absence (see Methods). Thus, it is our contention that the distribution maps in Figs. 6 and 7 accurately depict the high degree of exclusivity in the respective ranges of these species/lineages and their regional subclades.

Supplemental Appendix S2: Detailed analysis of *P. rugosus* and *P. barbatus* phylogeography

**Origins and evolution of *P. rugosus***

The *P. rugosus* samples in this study were recovered in a series of six successively branching clades. The two youngest groups in the Sonoran and Mojave deserts (Prug 2 and Prug 3, Fig. 3) are rooted by the next most basal group (Prug 1) on the Colorado Plateau, and that larger group is itself rooted by three more successive branches representing regional populations in the southern Sonoran-Sinaloan transition zone, the Vizcaino desert in the Baja Peninsula, and a corner of the southern Mexican Altiplano (S.Mx Prug, Baja Prug, and Basal Prug, Fig. 3). These three basal-most and southern-most clades were labeled as South Prug, but it is important to note that they do not form a monophyletic clade. Rather, they are potentially significant because their relative age and geographic positions suggest a specific route for *P. rugosus*’ ancestral dispersal and vicariance.

Following the generalized model of major vicariance events presented in Riddle and Hafner (2006), and the more specific patterns of southern vicariance suggested for *Crotalus polystictus*, *C. enyo*, and *C. cerastes* (Douglas et al. 2006), we can infer an early distribution of *P. rugosus* that spanned the Sierra Madre Occidental and the Pacific coast of Mexico prior to the opening of the Sea of Cortes. Such a model would place these initial vicariance events in the late Miocene to early Pliocene. First, the Basal Prug clade may have been isolated as a result of uplift or volcanic mountain building in the Sierra Madre Occidental (Van Devender 2000, Henry and Aranda-Gomez 2000). Then, the Baja Prug clade may have been isolated from the ancestral mainland distribution as a result of the progressive opening and widening of the Sea of Cortes, which began in the late Miocene and continued through the Pliocene (Riddle et al. 2000). The third basal lineage, S.Mx Prug, sits between these two other clades as the logical consequence of the hypothesized vicariance events to the east and west. However, the S.Mx Prug clade is the closest southern ancestor to all three of the younger *P. rugosus* clades in the north. This is a notably different pattern than what has been reported for a large number of mice and lizards (Riddle and Hafner 2006), birds (Zink et al. 2000), snakes (Devitt 2006, Castoe et al. 2007, Mulcahy 2008), and spiders (Crews and Hedin 2006). The majority of these studies have indicated similar basal divisions between the Chihuahuan desert, Sonoran desert, and the Baja peninsula, but they all differ markedly in that they show a consistent pattern of north-south continuity within those three corridors.

For example, most of these studies found evidence for a close relationship between populations in the northern Baja Peninsula and populations in southwestern California, with little or no signs of isolation between them. For taxa whose ranges also include the Sonoran and Mojave deserts, most of those studies have reported a sister relationship between the Peninsular/southern-California clades and the mainland clades that contact them near the Colorado River. Because of the frequency of this pattern, many authors have suggested that a series of marine incursions in the Pliocene and Pleistocene, known as the Bouse Embayment(s) and the San Gorgonio Constriction(s), may be responsible for creating and maintaining isolation between these largely parapatric distributions (Riddle and Hafner 2006, Crews and Hedin 2006, Devitt 2006). Alternatively, it is conceivable that these marine incursions only served to maintain isolation between groups that had previously diverged as a result of the earlier opening of the Sea of Cortes to the south. Because these two hypotheses invoke distinct geomorphological processes, presumably separated by a million years or more, one way to distinguish between them is by estimating the age of divergence between mainland and peninsular clades (Riddle et al. 2000, Crews and Hedin 2006). However, Grismer (1994) also suggested a number of other predictions that might be used to distinguish between a southern vicariance across the gulf in the Miocene, and a northern vicariance in the Pliocene. These points include an expectation that sister clades divided by the opening of the Sea of Cortes should be more southerly, and they should not contact one another in the north. These predictions have been supported by molecular evidence from several groups of *Crotalus* rattlesnakes (Douglas et al. 2006).

In contrast to the north-south continuity pattern described above, our Baja Prug sample is highly diverged from the broadly distributed Prug 3 clade that extends through southern California and into the northern edge of the Baja Peninsula (p-distance = 4.1%). Indeed, the closest Prug 3 population is only about 150 miles north of our Baja Prug sample, yet its mtDNA is more closely related to the distant populations of S.Mx Prug in mainland Mexico, and closer still to the Prug 2 and Prug 1 populations to the east (Figs. 3 & 5, Table 4). This suggests that the Baja and Prug 3 clades may have only recently come into their current proximate distributions, probably because of westward and southward expansion from the Prug 3 group after the aforementioned marine incursions retreated. The highly significant Fu's *Fs* statistic estimated for Prug 3 also supports a hypothesis of recent expansion for that clade (Table 6). Furthermore, the basal division between the southerly Baja Prug and S.Mx Prug clades is generally consistent with the predictions of a late Miocene transgulfian vicariance as outlined by Grismer (1994), as well as others (Riddle et al. 2000, Douglas et al. 2006).

The narrow distribution of contemporary *P. rugosus* along the Baja is another puzzling contrast to the broader distributions reported for other arid-adapted organisms with concurrent distributions in the southern Sonoran and Peninsular deserts. Unfortunately, this apparently narrow distribution limits our ability to reconstruct Baja Prug’s history within the rather detailed phylogeographic framework provided by several decades of intense study in other organisms (Riddle 2000, Zink et al. 2001, Lawlor et al. 2002, Murphy and Aguirre-Léon 2002). It is possible that the Baja Prug clade had previously existed in the north, and this haplotype may simply have been replaced by recent migration from the Prug 3 clade to the east. If this is the case, then larger population samples in this region may be able to detect signs of recent contact and introgression where this study’s broad sampling did not. Alternatively, it may be that the current Baja Prug sample is a remnant of an ancestral population that was restricted to the south by one or more of the seaways believed to have bisected the peninsula during the last 1-3 million years (Riddle and Hafner 2000, Crews and Hedin 2006). Regardless of these questions, this mtDNA pattern suggests that the ancestral Baja Prug clade has been isolated on the peninsula for most of the history of the extant *P. rugosus*, and it did not contribute its mitochondrial diversity to the evolution of the North Prug clades (Fig. 4).

The narrow distribution of the Basal Prug group is also markedly different from the predominant patterns reported for vertebrates (Zink et al. 2000, Riddle and Hafner 2006). In most cases, the clades in the southern limits of the Mexican Altiplano/Chihuahuan desert are closely related to lineages in the north, often extending into the Rio Grande Rift valley in New Mexico or onto the Colorado plateau (e.g. Riddle and Honeycutt 1990, Castoe et al. 2007, McGuire et al. 2007, Mulcahy 2008). As with the Baja Prug clade, this narrow distribution is somewhat puzzling because the northern Chihuahuan desert would seem to be a suitable habitat for an ancestral *P. rugosus* group, but we have recovered no evidence to indicate that the ancestral *P. rugosus* populations in the southern half of the Mexican Altiplano ever expanded northward into the core Chihuahuan desert. There is a vast distribution of *P. rugosus* at the northern edge of the Chihuahuan desert and on Colorado Plateau (Prug 1, Fig. 5), but it is more closely related to clades to the west of the continental divide in the Sonoran desert. We can only speculate as to the possible causes for the Basal Prug clade’s absence from the northern Chihuahuan desert, but it seems likely that a range of factors have contributed to its presently restricted distribution.

One possible explanation stems from the observation that all known Basal Prug populations sit just south of the Southern Coahuila Filter Barrier (SCFB), a boundary for the distributions of many mammalian species on the Mexican Altiplano (Baker 1956, Baker 1963, Petersen 1976). Like other hypothesized filter barriers, the SCFB is a somewhat indistinct boundary. It is formed by a combination of major drainage systems from the Sierra Madre Occidental in the west (chiefly the Río Nazas and the Río Aguanaval) and transverse extension of the Sierra Madre Oriental in the east (i.e., the Sierra de Parras) (Baker 1956, Hafner et al. 2008). This barrier appears less formidable at present, but extensive molecular evidence from pocket gophers suggests that it may have acted as a significant barrier in the Pleistocene, when pluvial cycles would have expanded flooding in the basins around the Río Nazas, creating lakes that persisted for years at a time (Hafner et al. 2008). The winged sexuals of *P. barbatus* and *P. rugosus* are presumably less affected by river barriers than terrestrial gophers, but plant fossils recovered from late Pleistocene packrat middens also indicate the presence of an extensive forest corridor that joined the upland fauna of Sierra Madre Occidental and Sierra Madre Oriental through this area (Betancourt et al. 1990). It is not known whether these forests were a constant feature of the early Pleistocene, nor is it clear how much they may have retracted during previous interglacials. However, it is likely that they created a significant ecological barrier to *P. barbatus* and *P. rugosus* dispersal when they were present. That said, the evidence for the SCFB does not seem to be a sufficient explanation for the limited distribution of the Basal Prug clade, especially within a broader hypothesis that suggests a Miocene to Pliocene age for the clade. We must therefore consider other factors that may have prevented, or replaced and obscured, this clade’s expansion(s) to the north. This includes the possibility of competition with both the hybrid H lineages to the north and the nearby *P. barbatus* clades, which occur in sympatry with the contemporary distribution of Basal Prug and partially surround it to the south (Fig. 4).

We have not detected colonies from the Basal Prug clade in sympatry with the *P. rugosus*-like H lineage, but the apparent dominance of the H lineage in the northern Chihuahuan desert, and the discovery of several H lineage populations to the south, suggests that these two clades may have come into contact at some point. The evidence for expansion in both the *P. barbatus* clades and the H lineage clades, but not in the Basal Prug clade, further suggests the possibility that the distributions reported here may be the result of recent range shifts. Indeed, it is conceivable that these distributions are not a stable configuration; this may only be a snapshot of what is actually an ongoing shift in the respective ranges of these groups. Conversely, it is also possible that the Basal Prug distribution is relatively stable, and the nexus of clades in this region may be the result of several largely independent histories. Additional details pertaining to the H lineages and *P. barbatus* clades will be discussed below. However, it bears mention that the region around the SCFB is as an intersection for at least five lineages, including three highly diverged clades of *P. barbatus* and *P. rugosus*, and the two hybrid H lineages that are probably derived from other contact zones in the north. Therefore, we suggest that there is a great need for both population level genetic analyses and detailed ecological study on the *Pogonomyrmex* populations in these areas.

S.Mx Prug is the youngest of the three South Prug clades, and it completes the atypical phylogeographic pattern for southern *P. rugosus* described above. Where the Basal Prug and Baja Prug clades are unusually isolated, being highly diverged from the *P. rugosus* clades to the north, the S.Mx Prug clade is unusual because it is the closest southern ancestor of the three widely distributed *P. rugosus* clades in the north. Therefore, S.Mx Prug appears to be something of a missing link between south and north, a pattern reflected not only in the phylogeny (Fig. 3), but also in the average genetic distances between clades (Table 4). The North Prug clades are all closer to the S.Mx Prug clade (average p-distances range from 3.1-3.7%), than to the Baja Prug and Basal Prug clades (average p-distances range from 4.1-4.8%). Thus, the geographic and phylogenetic position of the S.Mx Prug clade is important for our understanding of the larger phylogeographic history of the species.

It appears that the S.Mx Prug clade may have been the only southern *P. rugosus* group to expand north, through the nascent Sonoran and Mojave deserts, and onto the Colorado plateau. A dispersal pattern leading from the Sonoran-Sinaloan transition zone to the Colorado Plateau would be rather exceptional compared to the histories inferred for most arid-adapted vertebrates in this region. However, a number of vertebrate studies show a pattern of basal lineages in the Sonoran-Sinaloan region and a successive divergence of younger lineages to the north, especially through the Sonoran, Mojave, and Great Basin deserts. Such a pattern is known from several groups of snakes (Devitt 2006, Douglas et al. 2006, Mulcahy 2008), spiny lizards (Leaché and Mulcahy 2007), and flightless cactus beetles (Smith and Farrell 2005). This pattern is consistent with a broader desert flora and fauna hypothesis that traces the xeric adaptations of various desert species to tropical deciduous and thornscrub environments, such as those presently found in the Sonoran-Sinaloan transition zone, which may have been subject to periods of severe drought in the Miocene (Axelrod and Raven 1985, Van Devender 2000).

However, associating the S.Mx Prug clade with a proto-desert fauna hypothesis suggests that its distribution must date to at least the mid to late Miocene. Thus, it may be better to consider a less ancient and less dispersalist hypothesis, with the basal phylogenetic position of the S.Mx Prug clade being explained by isolation in an early Pleistocene refugia. Then the remaining three clades in the North Prug group could have fragmented during successive climatic shifts through the mid to late Pleistocene. The contemporary distribution of S.Mx Prug extends from the Río Fuerte in Sinaloa to areas just north of the Río Yaqui in Sonora, and the nearest Prug 2 population included in this study is just 50 miles to the north. Neither clade shows significant signs of recent population expansion according to our tests with Fu's *Fs* (Table 6), but they are both well supported as monophyletic groups (Fig. 3), so it is reasonable to assume that they have been evolving in allopatry for some time. Except for a few scattered populations from a congener (not shown here, but see *P. bicolor* in Cole 1968 and Johnson 2000a), there is very little to presently divide these two distributions, and it is quite possible that population genetic analyses might uncover evidence for more recent gene flow between them.

We are not aware of any specific hypotheses suggesting the Río Yaqui as a putative filter barrier during the Pleistocene, but it defines the southern margin of the Sonoran desert as defined by Shreve (1942). The river also defines the northern edge of the Sonoran-Sinaloan transition zone, which has been recognized as a significant boundary for a large number of arid-adapted rodent species (Hafner and Riddle 2005). The southern boundary of the S.Mx Prug clade coincides with the Río Fuerte and the Sierra Barabampo, which are generally recognized as the southern limit of the Sonoran-Sinaloan transition zone. The significance of the Río Fuerte area has also been supported by several phylogeographic studies on snakes, which identified it as the boundary between northern and southern sister species along the coast (Devitt 2006, Mulcahy 2008). Interestingly, the Río Fuerte-Sierra Barabampo boundary is also coincident with the northern limit of *Pogonomyrmex* *wheeleri*, which is a presumed sister species to *P. barbatus* and *P. rugosus*, and which dominates this narrow lowland corridor through most of Sinaloa and Nayarit (Cole 1968). *P. wheeleri* is apparently endemic to this area (Cole 1968, but see collections from Johnson 2000a), and its geographic position between these two sister species suggests a shared history for the group.

**Pleistocene fragmentation of the northern P. rugosus (ECD)**

Despite the uncertainty regarding their exact age, the cryptic fragmentation among seemingly contiguous distributions of northern *P. rugosus* suggests that even the youngest of these clades predate the modern day Holocene, an interglacial period that began approximately 11,000 years ago (Van Devender 2000). Thus, all three of the northern *P. rugosus* clades are likely to have undergone significant range contractions in the preceding Pleistocene epoch, when glacial and pluvial cycles led to long periods of decreased temperatures and increased rainfall, respectively. Although there are believed to have been as many as 15-20 glacial-interglacial cycles during the approximately 2.5 million years of the Pleistocene (Imbrie and Imbrie 1980), the majority of those cycles are almost completely unknown in continental fossil records, presumably because earlier events were eroded and overwritten by those that followed (Van Devender 2000). In the southwestern U.S. and Mexico, the best record of glacial-interglacial ecological shifts comes from thousands of packrat middens deposited over the last 40,000 years (Betancourt et al. 1990). These middens provide an abundance of pollen and seed fossils, allowing detailed reconstructions of plant community succession through the Last Glacial Maximum (LGM) and into the Holocene interglacial (Betancourt et al. 1990).

During the LGM, a combination of forest expansions and pluvial lakes restricted desert communities throughout most of the Basin and Range province and on the Colorado plateau (Spaulding et al. 1985, Betancourt et al. 1990, Thompson et al. 1993). However, only a few desert-like refugia have been identified in the midden record (e.g. Death Valley and the Lower Colorado River Valley (LCRV), so the exact locations and extent of forest free arid habitats during the LGM remains the subject of continued study and debate (Betancourt et al. 1990). Furthermore, it is unclear how the patterns from the last glacial-interglacial cycle relate to ecological shifts in the earlier Pleistocene and Pliocene, which were subject to somewhat different climate conditions and which may have been influenced by Plio-Pleistocene uplift in the Sierra Nevada range (Betancourt et al. 1990).

In light of these uncertainties, the early divergence of the Prug 1 clade among northern *P. rugosus* is especially interesting because its long term persistence on the Colorado Plateau suggests the presence of one or more previously unrecognized arid refugia in that area. Although we are not aware of any specific hypotheses for arid refugia on the plateau, their existence could also explain the persistence of a mid-early Pleistocene aged clade of arid-adapted grasshopper mice on the plateau (Riddle and Honeycutt 1990, Riddle 1995). In contrast, the majority of studies on similarly distributed desert taxa have found that extant populations on the plateau and in the Rio Grande Rift are relatively young, with evidence of recent expansion from the Chihuahuan Desert to the southeast (Jaeger et al. 2005, and Smith and Farrell 2005), the Mojave and Great Basin deserts to the west (Orange et al. 1999, Sinclair et al. 2004, Leaché and Mulcahy 2007), or both (Pook et al. 2000, McGuire et al. 2007, Mulcahy 2008). This view of recent, possibly post-Pleistocene, expansion onto the Colorado Plateau is generally consistent with midden-based reconstructions that depict widespread woodlands throughout the plateau and the Rio Grande Rift (Betancourt et al. 1990). However, Betancourt et al. also note that midden fossils are primarily representative of the rocky enclaves where they are found, so they do not necessarily reflect the ecological patterns in more open ground where *P. rugosus* colonies are likely to occur (1990).

The Prug 1 clade’s recent history is further complicated by its high level of internal fragmentation, which is evident in both its highly positive Fu's *Fs* statistic and in its long internal branching in the phylogeny (Table 6, Fig. 3). This fragmentation is notable because it is inconsistent with both hypotheses mentioned above. Recent colonization from outside the plateau and recent expansion from refugia on the plateau should both produce a similar pattern of decreased haplotype diversity. One obvious, albeit unlikely, explanation for this fragmentation could be that the ancestral *P. rugosus* populations on the plateau were simply resistant to the pressures of Pleistocene climate change; thus, they would not have experienced the genetic bottlenecking commensurate with a retreat to glacial refugia. However, such an explanation would run contrary to the well supported paradigm of arid range contractions during Pleistocene glacials, and it is also inconsistent with the multiple patterns of apparent Pleistocene range contraction in several lower elevation regional clades of *P. rugosus* and *P. barbatus* (discussed below).

Therefore, we hypothesize that there may be multiple distinct subgroups nested within our nominal Prug 1 clade, each derived from its own discrete refugium. This second hypothesis is supported by a closer examination of the internal structure within Prug 1, which reveals three geographic clusters of highly similar haplotypes with very low internal divergence (p-distances range from 0.1% to 0.46%), and much higher divergences between the three groups (ranging from 1.1% to 2.97%). Interestingly, the most diverged of these three groups is composed of just three populations sampled from the Rio Grande Rift in New Mexico (38RUG, 39RUG, and 43 RUG; Fig. 3 and Table 1). The two more closely related groups within Prug 1 include the nine populations on the plateau and one at the northern end of the Rio Grande Rift. The significance of this pattern, including its indications for multiple microrefugia on the plateau and in the Rio Grande Rift, remains unclear. However, Riddle and Honeycutt offered a similar explanation for the surprising genetic diversity in their grasshopper mice samples from the plateau (1990), and most of the phylogeographic evidence for recent colonization of the plateau comes from herpetofauna studies. Therefore, additional genetic studies of both *P. rugosus* and a diverse range of other taxa in this region seem warranted.

It bears mentioning that, although the inter-regional relationships for northern *P. rugosus* seem to be somewhat novel, they do not necessarily suggest novel routes of dispersal between the Sonoran and Mojave deserts and the Colorado Plateau. Rather, it is likely that the *P. rugosus* on the plateau are derived from similar expansions to what is known from the various reptile and amphibian species cited above, but the direction of these ancestral dispersal events has been obscured in *P. rugosus* by more recent Pleistocene processes. Indeed, it is quite possible that these other arid species had Pliocene or early Pleistocene distributions on the plateau concurrent with the initial *P. rugosus* colonization, and it is only the uncommon persistence of the Prug 1 clade through the Pleistocene glacial periods that gives the appearance of a dramatically reshaped phylogeographic history. Thus, where the linear inter-regional relationships indicated in other studies may suggest dispersal (e.g., Smith and Farrell 2005), the reflexive patterns in northern *P. rugosus* suggest a single broad distribution that was fragmented into progressively smaller partitions over time.

The complex reflexive patterns observed in the northern *P. rugosus* (Fig. 6) are probably the result of successive climate shifts that broke off peripheral portions of the ancestral distribution in the south (S.Mx Prug), then in the north (Prug 1), and then finally breaking apart the youngest clades in the center (Prug 2 and Prug 3). These reflexive or nested histories seem to be a common feature of finer scale phylogeographic analyses in the Sonoran and Mojave deserts, and often with a basal clade in southern Sonora (Douglas et al. 2006, Leaché and Mulcahy 2007, Leavitt et al. 2007, Mulcahy 2008).

Setting their unusual phylogenetic rooting aside, the geographic positions of the Prug 2 and Prug 3 clades are consistent with a common phylogeographic pattern of Pleistocene isolation between western/Mojave/ Lower Colorado River Valley (LCRV) and eastern/Sonoran clades (e.g. Riddle 1995, Jaeger et al. 2005, Douglas et al. 2006, McGuire et al. 2006, Castoe et al. 2007, Leaché and Mulcahy 2007, Leavitt et al. 2007, Mulcahy 2008, Jezkova et al. 2009). This pattern is also consistent with midden evidence that suggests Mojave-like refugia in the LCRV and Sonoran Desert refugia somewhere in central Sonora, Mexico (Betancourt et al. 1990). Although these studies illustrate a degree of broad conformity among taxonomically diverse samples, they also reveal extensive inter-taxa variability in the demarcation of modern distribution boundaries. In *P. rugosus*, it appears that the western/Mojave/LCRV clade (Prug 3) has undergone at least one large range expansion in either late Pleistocene or Holocene time, as indicated by the large geographic distances between similar haplotypes and the statistically significant estimates for Fu's *Fs* and *R2* (Figs. 3 & 5, Table 6). In contrast, the Prug 2 clade (which occupies the Sonoran desert south of the Gila River) produced Fu's *Fs* and *R2* estimates that were not quite significant, suggesting that it may not have experienced a major expansion in recent Pleistocene/Holocene time (Table 6). This difference may imply that the Prug 2 clade’s distribution in the Sonoran desert has been relatively stable during late Pleistocene climate shifts, which has also been suggested for a similarly distributed clade of desert pocket mice (Jezkova et al. 2009). However, the same caveat as was discussed for Prug 1 applies (i.e., the genetic signature of range expansion in Prug 2 may be obscured because the clade contains diversity from multiple discrete subgroups).

Interestingly, there is also evidence for the northward expansion of tropical habitats in the LCRV and other riparian corridors during some earlier interglacial cycles of the Pleistocene (Van Devender 2000). Although less complete, these earlier Pleistocene records are interesting because they demonstrate the degree to which ecological and climatological patterns during earlier glacial-interglacial cycles may have varied from the comparatively well understood patterns of the LGM and Holocene. More specifically, intermittent tropical expansions may have displaced or limited arid species distributions during interglacials, even as the glacial forests were retreating.

The hybrid J1 lineage is also a member of the northern *P. rugosus* mtDNA species tree (Fig. 3 this paper; also Helms Cahan and Keller 2003, Anderson et al. 2006, Schwander et al. 2007a). Although our phylogenetic results were somewhat ambiguous regarding the branching order of Prug 2, Prug 3, and J1 (see Results), we can conclude that all three clades shared a common maternal ancestor sometime in the mid to late Pleistocene. However, the unique characteristics of the hybrid J1 lineage, especially its mutualistic dependence on mating—and therefore sympatry—with the J2 lineage, suggest that it should be considered alongside the J2 lineage and regarded as distinct from the remainder of *P. rugosus* (Cahan and Keller 2003, Anderson et al. 2006, Schwander et al. 2007a). Thus, the overlapping distributions of the J1 and J2 lineages in the Apache Highlands Ecoregion will be discussed below, along with the related H1/H2 lineage pair.

**Origins and evolution of *P. barbatus***

Like the *P. rugosus* patterns described above, the history of extant *P. barbatus* seems to have been shaped by a combination of early inter-regional divisions and more recent Pleistocene contraction, fragmentation, and subsequent expansion. However, where the *P. rugosus* subtree reflects a single ladderized history of subclade divergence, the *P. barbatus* tree splits into four branches at its most basal edge (Fig. 3). Three of these four basal clades are found in the southern Mexican Altiplano, which suggests that this region may be the ancestral source for *P. barbatus* radiations. Two of these basal clades appear to be relicts: they are both known from just two populations each (i.e., four total), and our sampling in that area suggests that they may both be limited to narrow distributions along the western margins of the Sierra Madre Oriental (Basal Pbar North and Basal Pbar South, Figs. 3 & 5). Although they are not supported as a monophyletic clade, we have lumped them together as the nominal Basal Pbar group because of their relictual and geographic similarities.

In contrast, the other two basal clades have been highly successful, evolving in parallel through what seems to be an old and surprisingly broad East-West division in *P. barbatus* (Figs. 3 & 4, East Pbar clade vs. the clade containing both SWest Pbar and the J2 & H lineages). These two largest clades are divided into three macro groups, and those macro groups are further divided into six major subgroups that correspond to discrete geographic distributions (SWest Pbar 1, SWest Pbar 2, J2, H1 & H2, East Pbar 1, and East Pbar 2). If we only consider the younger nodes of the mtDNA phylogeny (i.e., within each macro group), these subgroup distributions are generally consistent with established biogeographic regions and Pleistocene fragmentation patterns observed in other taxa (detailed below). However, the deeper nodes of the *P. barbatus* subtree (i.e., among macro groups) reveal an older and broader phylogeographic history that is much more complex. This complexity stems from the apparent incongruities between the modern distributions of these three macro groups, which meet in broad parapatry (or sometimes sympatry) near the center of the Chihuahuan Desert, and the necessary inferences for a succession of ancient dispersal and vicariance events through that same area. To reconcile their modern distributions with the deeper geographic relationships indicated in the phylogeny, we hypothesize a layered, reflexive history for *P. barbatus*-derived lineages in the northern Chihuahuan Desert.

At present, the northern Chihuahuan Desert is inhabited by two distantly related subgroups. The hybrid H lineages with GCD dominate most of the northern Mexican Altiplano, and their distribution extends north into the Intermontane Plateaus of the U.S. and as far south as Aguascalientes in central Mexico. The East Pbar 1 clade inhabits the eastern margins of the northern Chihuahuan Desert from the Bolsón de Mapimí in the south through the southern Intermontane Plateaus and plains in the U.S. Despite the broad overlap between these two subgroups, and contrary to the implicit assumption of most previous molecular studies on the GCD system, our data indicates that the ECD *P. barbatus* found in New Mexico and Texas (i.e., the East Pbar) are not a direct maternal ancestor to the hybrid H lineages. Rather, the H lineages clade and the East Pbar 1 clade, which together form the geographic center of the *P. barbatus* mtDNA-species distribution, are each most closely related to peripheral populations outside the Chihuahuan Desert.

**Phylogeographic position of the J and H lineages within P. barbatus**

Our mtDNA phylogeny indicates that the broadly distributed H lineages are either sister to – or nested within – the J2 clade, which sits just outside the northwestern edge of the Chihuahuan Desert in the Apache Highlands Ecoregion (Fig. 7). The J2 clade is itself rooted by both the MX2 sample and the larger SWest Pbar clade from central Mexico (Fig. 3). This means that the *P. barbatus*-like J2 lineage is conspicuously removed from its closest *P. barbatus* ancestors, with at least 500 miles of Chihuahuan Desert (1,000 miles for MX2) between them, and the intervening area is now dominated by the hybrid H lineages.

Within a single species, this pattern could be explained by nested fragmentation, with the common ancestor of J and H establishing a broad distribution, breaking away from the southern *P. barbatus*, and then later breaking apart into southeastern (H) and northwestern (J) subclades. However, the hybrid character of the H lineages necessarily excludes a hypothesis of fragmentation because, by definition, they are not derived from a single broadly distributed ancestor but rather from two separate species that met across a contact zone. Moreover, the H lineages’ hybrid mtDNA can be traced to a single origin (i.e., they form a monophyletic clade), which means that the hybrid exchange either occurred in a relatively narrow space or it was bottlenecked sometime after. Thus, any populations that carry this hybrid signature must be the result of proliferation and expansion from this effectively discrete introgression event.

Having ruled out fragmentation, this phylogeographic pattern indicates there must have been a historical corridor of *P. barbatus* between the southern Mexican Altiplano and the Apache Highlands, and the most likely route between these regions is through the northern Chihuahuan Desert where the H lineages now dominate. The modern J1/J2 and H1/H2 distributions meet in the narrow, arid-lowlands corridor known to biogeographers as either the Deming Plains or the Cochise Filter Barrier (CFB) (Morafka 1977). This area – which is broken by a series of sky islands and bounded in the north and south by the Mogollon Rim and the Sierra Madre Occidental, respectively – is believed to be the only arid corridor between eastern and western deserts after Miocene-Pliocene uplift in the aforementioned mountain ranges (Riddle and Hafner 2006b, Pyron and Burbrink 2009). This suggests that the hybridization events that gave rise to the H lineages occurred somewhere along the northwestern edge of the Chihuahuan Desert, probably in the vicinity of the CFB, and the breadth of their modern distribution must therefore be the result of an extensive expansion from that area. The Fu’s *Fs* estimate for the combined H lineages may also be indicative of recent expansion in this group (Table 6).

Consequently, we hypothesize a succession of dispersal events through the historical Chihuahuan Desert: First, the ancestral *P. barbatus* must have expanded north, founding populations in the vicinity of the CFB that would eventually hybridize with *P. rugosus* to create the H1, H2, and J1 lineages. At some point later, these western *P. barbatus* retreated from the northern Chihuahuan Desert. Then, either concurrently with or subsequent to this retreat by *P. barbatus*, the hybrid H lineages expanded outward and carried *P. barbatus*-derived haplotypes back south through the Chihuahuan Desert, as well as east and north through arid lands in the U.S.

**Eastern P. barbatus**

Similar to the J2 & H group, the East Pbar clade can be roughly divided into interior (East Pbar 1) and peripheral (East Pbar 2) sister clades (Fig. 5). Our mtDNA phylogeny indicates that the East Pbar group is a long-diverged sister to all other *P. barbatus* (which are rooted on the southern altiplano of Mexico), but like the J2 lineage, it seems that the earlier patterns of dispersal and vicariance for the East Pbar have largely been obscured by more recent events. The deep divergence of the East Pbar suggests that it was isolated in the late Miocene or Pliocene (approximately 3-4 mya per the 1.5% calibration from Quek et al. 2004, Table 3), but the physiographic and/or ecological causes of this vicariance are unclear.

The distribution of the modern East Pbar 1 clade is consistent with several hypotheses for Pleistocene vicariance in the Chihuahuan Desert. First, our limited sample of southerly East Pbar 1 populations (Pb419, Pb457, Pr425; Table 1) are consistent with the well supported hypothesis for one or more Pleistocene refugia in the Bolsón de Mapimí (Morafka 1977, Elias et al. 1995, Orange et al. 1999, Riddle and Hafner 2006b, Castoe et al. 2007, but see Van Devender et al. 1985). Second, these southernmost East Pbar 1 populations are notably diverged from their more northern cousins in New Mexico and Texas (Fig. 3), which supports a hypothesis for one or more Chihuahuan Desert refugia north of the Rio Grande River (Smith and Farrell 2005). Third, the division between East Pbar 1 and East Pbar 2 is consistent with an emerging pattern of Pleistocene vicariance between interior and Gulf Coast clades in snakes (Castoe et al. 2007, Mulcahy 2008). Finally, the East Pbar 1 and SWest Pbar clades appear to meet along the above-mentioned Southern Coahuila Filter Barrier (Hafner et al. 2008), which further suggests that *Pogonomyrmex* species may have been affected by the SCFB and other ecological drivers of sub-province fragmentation in the Chihuahuan Desert (Morafka 1977). However, the chronology of major divergence events recovered in our phylogeny suggests that a Pleistocene-aged SCFB could not have been the initial cause of divergence between the East Pbar macro group and its more western sisters. Rather, the SCFB may have only served to maintain the East Pbar 1 group’s isolation from its southern cousins.

As detailed above, the phylogenetic relationship between the J2 and SWest Pbar clades indicates that there was likely a corridor of western *P. barbatus* on the northern altiplano long after the East Pbar group’s mitochondrial lineage had diverged from the rest of *P. barbatus* (Fig. 7). This chronology leads us to speculate that the East Pbar 1 clade may have been absent from the northern altiplano during the Pliocene and early Pleistocene, and it may be that they only colonized the Bolsón de Mapimí after the western *P. barbatus* clades retreated from that area. This hypothesis also suggests a possible mechanism for the earlier vicariance of the East Pbar clade. If we assume that the East Pbar 1 distribution formed after dispersal from a common ancestor with East Pbar 2, then it follows that the East Pbar group may have initially evolved as a coastal isolate, separated from the western *P. barbatus* by the Sierra Madre Oriental.

Regardless of their deeper origins, there is strong evidence to suggest that the East Pbar 2 clade has experienced an exceptionally broad expansion in recent history. In addition to its highly significant Fu's *Fs* (Table 6), the East Pbar 2 clade includes highly similar haplotypes (i.e., 0.1-1.0% p-distance) drawn from populations >950 miles apart. The Gulf/Tamaulipan Coastal Plains are a transitional semi-arid shrub province (Brown et al. 2007), and a number of the resident vertebrate species are closely related to populations in the arid grasslands and deserts to the north and west (Riddle and Honeycutt 1990, Castoe et al. 2007). However, most phylogeographic studies on related taxa have included little or no sampling south of Texas, and we know of only one other phylogeographic study that has found evidence of recent expansion, and thus presumed Pleistocene contraction, through this corridor (Mulcahy 2008). Interestingly, Mulcahy (2008) found evidence for a cryptic mtDNA division in the Great Plains distribution of nightsnakes that mirrors the pattern reported here for East Pbar 2. In both cases, easterly populations in the U.S. were found to be more closely related to geographically-distant Mexican populations in Tamaulipas than to nearby populations to the west (see East Pbar 1 and East Pbar 2, Fig. 5). Mulcahy argues that this pattern is indicative of a northward expansion from a Tamaulipan refugium (2008), but the molecular data presented here do not provide any indication for an origin or direction for the hypothesized expansion in East Pbar 2.

**Southwestern and Basal *P. barbatus***

Of all the biogeographic provinces inhabited by *Pogonomyrmex barbatus* and *P. rugosus*, the southern Mexican Altiplano is the most fragmented. In addition to the entire Basal Prug distribution and incursions from the H lineages and East Pbar 1 from the north, the southern Mexican Altiplano also harbors the two highly diverged Basal Pbar clades in the east, and it is dominated by the two sister clades of SWest Pbar in its center and western margins (Fig. 5). This exceptional concentration of mtDNA diversity within a single physiographic region and biogeographic province begs a number of questions as to the mechanisms that have arrayed, and apparently maintained, so many discrete clades in broad regional parapatry. Unfortunately, there is a notable paucity of sampling and phylogeographic study on arid-adapted species in this region, and we are unaware of any clear indications for historical barriers that might be geographically coincident with these clades’ current borders.

The first hypothesis is supported by the observation that four of the seven clades found in the southern altiplano extend outside its geographic boundaries, and three of those clades show signs of recent expansion. SWest Pbar 1 and East Pbar 1 were significant according to Fu’s *Fs* (Table 6), and we have inferred expansion in the H lineages as well (see above). This suggests that several of the most widely distributed clades in this region, chief among them SWest Pbar 1, may have diverged in allopatry outside the traditional boundaries of the southern Mexican Altiplano (i.e., the plateau bounded by the southern Sierra Madre Occidental, Sierra Madre Oriental, and the Trans Mexican Volcanic Belt (TMVB)). This region’s nominal southern boundary, the TMVB, is not a range of continuous high ridges like the Sierra Madres. Rather, it is a somewhat porous string of sky-island peaks, more akin to the sky-island ranges that dot the lowland Cochise Filter Barrier area between the Sierra Madre Occidental and the Mogollon Rim. The contemporary SWest Pbar 1 distribution spans the TMVB with closely related populations to the north and south (Fig. 5), which suggests that it is not currently a barrier to *P. barbatus*. However, there is considerable geological, paleoclimatic, and phylogeographic evidence to suggest that the TMVB and its surrounding environs have experienced major perturbations over the last several million years.

The TMVB is believed to have originated in the mid-to-late Miocene (Ferrari et al. 1999, Ferrari et al. 2000) and there are indications that extensive volcanic activity may have persisted in the region throughout the Pliocene, Pleistocene, and Holocene (Johnson and Harrison 1990, Demastes et al. 2002). Similar to the northern deserts, it is believed that Pleistocene climate fluctuations led to repeated expansions and contractions of the pine-oak woodlands around the highlands of the southern altiplano and TMVB (Metcalfe 2006, Gugger et al. 2011). A large number of phylogeographic studies indicate that these range expansions were broad enough to temporarily unite the distributions of woodland/montane species of plants, insects, and vertebrates across the southern altiplano, throughout the TMVB, and among the three ranges of the Sierra Madre (e.g., Sullivan et al. 1997, Demastes et al. 2001, Anducho-Reyes et al. 2008, McCormack et al. 2008, Bryson et al. 2011). These taxa now dominate highland regions where *P. barbatus* does not seem to occur, so we can assume that their Pleistocene expansions across lower elevations were likely to have coincided with contractions in the distributions of *P. barbatus*. Many of these studies identified patterns of mid-Pleistocene vicariance around the TMVB, which would be consistent with our coarse estimate of divergence between SWest Pbar 1 and SWest Pbar 2 (1.78 mya, Table 4). This region’s complex topography and fragmented ecology may also have contributed to the apparent isolation of the lone MX2 sample (Fig. 5), but further speculation on that sample’s phylogeographic history must wait for additional sampling in that area.

Overall, the evidence above seems to support our first hypothesis: The mtDNA fragmentation and broad parapatry on the southern altiplano may primarily be the result of recent contact among sister clades that diverged in allopatry, i.e., Pleistocene refugia. However, the SWest Pbar 2 and Basal Pbar clades do not match this pattern. They show no signs of recent expansion according to Fu’s *Fs*, and their contemporary distributions suggest that, if they had been isolated during the Pleistocene, they might well have been isolated within the same arid refugia. Populations from the two clades of the nominal Basal Pbar group, along with SWest Pbar 2, were all discovered in close proximity (i.e., 40-90 miles apart) along the western margins of the Sierra Madre Oriental (Fig. 5). However, the deep mtDNA divergences between these three clades suggest a possible Pliocene divergence (2.63-3.23 mya, Table 4).

It seems unlikely that either distance or ephemeral barriers alone could have maintained these narrowly distributed Basal Pbar clades over such an extended period, but we cannot assess alternative hypotheses, such as speciation, without further study on their ecology and genetics. Such study may be especially warranted with the two northern populations in the Basal Pbar group (Pr445 and Pr451, Table 1), which are particularly interesting because they possess a distinctly *P. rugosus*-like morphology. They are nominally classified here as *P. barbatus* because their mtDNA sequences were strongly supported as members of the *P. barbatus* subtree (see Results), but their basal position in the phylogenetic tree suggests that their incongruent mtDNA-morphology pattern could be the result of either incomplete lineage sorting or ancient introgression. Future research with nuclear DNA markers could indicate whether these populations are a basal offshoot of either species, or they may even warrant classification as a plesiomorphic third species. In any case, the central phylogenetic and geographic positions of these Basal Pbar populations suggest that they may be an important resource for studies on the origins of the greater *P. barbatus* and *P. rugosus* species complex.

References

Alexander Pyron, R., and F. T. Burbrink. 2009. Lineage diversification in a widespread species: roles for niche divergence and conservatism in the common kingsnake, Lampropeltis getula. Molecular ecology 18:3443-57. doi: 10.1111/j.1365-294X.2009.04292.x.

Anducho-Reyes, M. a, A. I. Cognato, J. L. Hayes, and G. Zúñiga. 2008. Phylogeography of the bark beetle Dendroctonus mexicanus Hopkins (Coleoptera: Curculionidae: Scolytinae). Molecular phylogenetics and evolution 49:930-40. Elsevier Inc. doi: 10.1016/j.ympev.2008.09.005.

Axelrod, D. I., and P. H. Raven. 1985. Origins of the Cordilleran Flora. Journal of Biogeography 12:21-47. Blackwell Publishing. Retrieved from http://www.jstor.org/stable/2845027.

Baker, R. H. 1963. Geographical Distribution of Terrestrial Mammals in Middle America. American Midland Naturalist 70:208-249. The University of Notre Dame. Retrieved from http://www.jstor.org/stable/2422784.

Bryson, R. W., R. W. Murphy, A. Lathrop, and D. Lazcano-Villareal. 2011. Evolutionary drivers of phylogeographical diversity in the highlands of Mexico: a case study of the Crotalus triseriatus species group of montane rattlesnakes. Journal of Biogeography 38:697-710. doi: 10.1111/j.1365-2699.2010.02431.x.

Demastes, J. W., T. a Spradling, M. S. Hafner, D. J. Hafner, and D. L. Reed. 2002. Systematics and phylogeography of pocket gophers in the genera Cratogeomys and Pappogeomys. Molecular phylogenetics and evolution 22:144-54. doi: 10.1006/mpev.2001.1044.

Ferrari, L., S. A. Alaniz-alvarez, G. L.-hernández I. D. Geología, U. Autónoma, and D. S. Luis. 1999. Variation of Cenozoic extension and volcanism across the southern Sierra Madre Occidental volcanic province, Mexico:347-363.

Ferrari, L., S. Conticelli, G. Vaggelli, C. M. Petrone, and P. Manetti. 2000. Late Miocene volcanism and intra-arc tectonics during the early development of the Trans-Mexican Volcanic Belt. Tectonophysics 318:161-185. doi: 10.1016/S0040-1951(99)00310-8.

Grismer, L. L. 2000. Evolutionary biogeography on Mexico ’ s Baja California peninsula : A synthesis of molecules and historical geology 97:14017-14018.

Gugger, P. F., A. González-Rodríguez, H. Rodríguez-Correa, S. Sugita, and J. Cavender-Bares. 2011. Southward Pleistocene migration of Douglas-fir into Mexico: phylogeography, ecological niche modeling, and conservation of “rear edge” populations. The New phytologist 189:1185-99. doi: 10.1111/j.1469-8137.2010.03559.x.

Henry, C. D., and J. J. Aranda-gomez. 2000. Plate interactions control middle – late Miocene , proto-Gulf and Basin and Range extension in the southern Basin and Range 318:1-26.

Imbrie, J., and J. Z. Imbrie. 1980. Modeling the climatic response to orbital variations. Science (New York, N.Y.) 207:943-53. doi: 10.1126/science.207.4434.943.

Johnson, C. A., and C. G. A. Harrison. 1990. Neotectonics in central Mexico. Physics of the Earth and Planetary Interiors 64:187-210. doi: 10.1016/0031-9201(90)90037-X.

McCormack, J. E., a T. Peterson, E. Bonaccorso, and T. B. Smith. 2008. Speciation in the highlands of Mexico: genetic and phenotypic divergence in the Mexican jay (Aphelocoma ultramarina). Molecular ecology 17:2505-21. doi: 10.1111/j.1365-294X.2008.03776.x.

Metcalfe, S. E. 2006. Late quaternary environments of the northern deserts and central transvolcanic belt of Mexico. 93:258-273.

Petersen, M. K., T. R. Nazas, M. Distribution, M. K. P. Source, T. S. Naturalist, and N. S. Url. 1976. The Rio Nazas as a Factor in Mammalian Distribution in Durango, Mexico. The Southwestern Naturalist 20:495-502. Southwestern Association of Naturalists. Retrieved from http://www.jstor.org/stable/3669866.

Pook, C. E., W. Wüster, and R. S. Thorpe. 2000. Historical biogeography of the Western Rattlesnake (Serpentes: viperidae: Crotalus viridis), inferred from mitochondrial DNA sequence information. Molecular phylogenetics and evolution 15:269-82. doi: 10.1006/mpev.1999.0756.

Riddle, B. R., and D. Hafner. 2000. Cryptic vicariance in the historical assembly of a Baja California Peninsular Desert biota. Proceedings of the National Academy of Sciences 97:14438-14443. Retrieved June 28, 2012, from http://www.pnas.org/content/97/26/14438.short.

Sinclair, E. a, R. L. Bezy, K. Bolles, J. L. Camarillo, K. a Crandall, and J. W. Sites. 2004. Testing species boundaries in an ancient species complex with deep phylogeographic history: genus Xantusia (Squamata: Xantusiidae). The American naturalist 164:396-414. doi: 10.1086/381404.

Sullivan, J., J. a Markert, and C. W. Kilpatrick. 1997. Phylogeography and molecular systematics of the Peromyscus aztecus species group (Rodentia: Muridae) inferred using parsimony and likelihood. Systematic biology 46:426-40. Retrieved from http://www.ncbi.nlm.nih.gov/pubmed/11975329.

Zink, R., A. Kessen, T. Line, and R. C. Blackwell-rago. 2001. Comparative phylogeography of some aridland bird species. The Condor 103:1-10. Retrieved June 28, 2012, from http://www.bioone.org/doi/abs/10.1650/0010-5422(2001)103%5B0001:CPOSAB%5D2.0.CO%3B2.
